# Insights on the neurocognitive mechanisms underlying hippocampus-dependent memory impairment in COVID-19

**DOI:** 10.1038/s41598-025-04166-2

**Published:** 2025-06-20

**Authors:** Patric Meyer, Ann-Kathrin Zaiser

**Affiliations:** 1 Cognitive Neuroscience Unit, School of Psychology, SRH University of Applied Sciences Heidelberg, Maria-Probst-Strasse 3, D-69123 Heidelberg, Germany; 2 Department for General and Applied Linguistics, Ruprecht-Karls-University Heidelberg, Heidelberg, Germany; 3 Network Aging Research (NAR), Ruprecht-Karls-University Heidelberg, Heidelberg, Germany

**Keywords:** COVID-19, Cognitive impairment, Pattern separation, Neurogenesis, Hippocampus, Cognitive neuroscience, Viral infection, Human behaviour

## Abstract

COVID-19 has been linked to acute and long-term cognitive impairments, including memory and concentration deficits, as well as neuropsychiatric symptoms such as anxiety and depression. However, the neuropathophysiological mechanisms underlying these cognitive and affective changes remain poorly understood. Accumulating evidence points towards neuroinflammation as a potential driver of most acute and post-acute neurofunctional symptoms. In this study, we aimed to comprehensively characterize cognitive impairment associated with COVID-19 using a large online cohort of over 1400 participants, including individuals reporting a previous SARS-CoV-2 infection and individuals who had never been tested positive. Our cognitive test battery covered alertness, executive functions, and episodic long-term memory. Our results demonstrate a pronounced and selective impairment of individuals previously infected in a mnemonic discrimination task known to engage hippocampus-dependent pattern separation. This impairment remained statistically significant after controlling for potential confounding factors (i.e., age, gender, education, depressiveness, anxiety, and stress). This finding, derived indirectly from behavioral performance, suggests compromised hippocampal neurogenesis following infection, which may contribute to COVID-related memory deficits. Our study has important implications for understanding the neurofunctional consequences of COVID-19 and highlights the potential significance of neuroinflammation in the manifestation of cognitive impairments.

**Supplementary Information:**

The online version contains supplementary material available at 10.1038/s41598-025-04166-2.

## Introduction

The COVID-19 pandemic has unleashed widespread health, economic, and social disruptions, leaving no corner of the globe untouched by its impact. Beyond the well-known acute respiratory symptoms, infection with the SARS-CoV-2 virus has been linked to a broad spectrum of neurological manifestations as well as neuropsychiatric symptoms that emerge during the acute phase and may persist, resulting in long-term residual effects. Among them are anxiety, depression, and posttraumatic stress disorder (PTSD) symptoms, insomnia, to name a few^1–4^. COVID-19 survivors also commonly grapple with a range of cognitive symptoms, affecting various domains such as memory, attention, and concentration^5,6^. These symptoms have been reported in a significant number of patients, profoundly impacting their daily lives and overall quality of life^7^. Despite early focus on respiratory and cardiovascular complications, recognition of the neuropsychiatric and cognitive sequelae has grown due to their impact on patients’ well-being and functionality. As it is known that COVID symptoms can persist well beyond the acute phase of COVID-19 infection^8,9^, a comprehensive understanding of the nature of those neuropsychiatric and cognitive manifestations is essential for effective diagnosis, management, and patient care.

Recent findings have begun to shed light on those post-acute sequelae of COVID-19, revealing a spectrum of persistent cognitive symptoms. Numerous studies have traditionally relied on questionnaires based on patients’ subjective self-reports^10^. However, there is often a discrepancy between subjective complaints and objective test results gained from experimental data, with approximately 40% of patients showing this inconsistency^11^. More recent investigations have taken a different approach by utilizing direct experimental testing of patients’ cognitive functions to document a diverse array of post-COVID-19 cognitive deficits. Specifically, these studies have revealed particular impairments in attention, immediate and delayed verbal memory, learning^12,13^, and executive functions^5,6,14,15^.

Remarkably, these cognitive symptoms have been observed to persist for at least one year after discharge^16^. Despite this growing understanding, the factors influencing those deficits, and their neurobiological underpinnings, remain largely unclear, likely due to the complexity of the observable symptoms, which suggests a diverse and interrelated causality. However, unraveling the precise mechanisms behind these symptoms as well as a more profound comprehension of their neurobiological bases is crucial for devising targeted interventions, improving patient care, restoring functionality, and enhancing the quality of life for those affected by post-COVID-19 syndrome, that is, persisting symptoms at least 12 weeks after the start of acute COVID-19^17^. Particularly impairments in memory, executive functions, and attention, can substantially hinder an individual’s ability to perform routine tasks, engage in work or education, and maintain social relationships^18–20^.

The variability in findings across studies investigating memory impairments in COVID-19 is considerable, with differences in age, sex, severity of illness, and time since infection among participants^21,22^. Studies have included both hospitalized and non-hospitalized patients, with some studies encompassing both groups^15,23,24^. Among hospitalized patients, the severity of the condition varies, with differences in the need for intensive-care treatment, oxygen therapy, endotracheal intubation, mechanical ventilation, and other medical interventions, as well as in the average length of hospitalization. Additionally, the time elapsed between COVID-19 infection and assessment varies among studies. Various methods and tests are used to assess memory, with some studies including control groups while others do not (see^25^, for an overview). In their systematic review, Llana et al.^25^ examined the existing evidence on objective memory impairments in post-COVID-19 syndrome, considering sample and study design characteristics, as well as associations between memory performance and epidemiological, clinical, and pathological features. Poor verbal learning was reported (in 6–58% of participants, depending on the study), followed by deficits in long-term (4–58%) and short-term (4–37%) verbal memory. The visuo-spatial component of memory was studied less than the verbal component, showing impairment of long-term retention of visual items (10–49%). Moreover, COVID-19 severity in the acute stage was not systematically associated with poor memory performance^25^.

Neuroimaging studies on post-acute COVID-19 patients have provided some insights into the impact of the virus on structural and functional alterations in both cortical and subcortical brain regions^26–28^. Specifically, the hippocampus has demonstrated notable structural as well as functional changes at 4-month and 6-month follow-up intervals^27,29^ but alterations have also been observed already since the acute and post-acute phases^30–32^. These findings indicate that the hippocampus may be particularly vulnerable to COVID-19. Both diffusion tensor imaging (DTI) and magnetic resonance imaging (MRI) investigations have demonstrated abnormal microstructural changes and atrophy in the hippocampus of COVID-19 survivors, with these alterations closely associated with memory deficits and cognitive impairments^31,33^. In an investigation employing MRI on a COVID-19 patient, significant hyperintensities were detected in the unilateral ventricle and temporal lobe, accompanied by hippocampal atrophy^34^. In this context, MRI refers to structural imaging techniques, such as T1-weighted volumetric sequences, which are used to assess grey matter volume and overall brain morphology. DTI, a specific MRI-based technique, allows for the analysis of white matter microstructure by measuring the diffusion of water molecules along axonal pathways. Together, these modalities provide complementary insights into both structural and microstructural changes in the brain.

The hippocampus, located deep within the medial temporal lobe, plays a critical role in memory and cognitive functions, particularly in learning and spatial processing^35^. Damage to this region has been consistently associated with memory impairments and cognitive deficits in a wide range of neurological and psychiatric disorders^36^. A distinct subregion within the hippocampus, the dentate gyrus (DG), plays a critical role in information processing and integration. Positioned in the anterior portion of the hippocampus, the DG exhibits a distinctive granule cell layer characterized by densely packed granule cells^37^. It receives input from the entorhinal cortex via the perforant pathway and projects its output to the CA3 region of the hippocampus through mossy fibers, forming a unidirectional connectivity vital for hippocampal circuitry^38^. As a “gatekeeper” for memory formation, the DG is actively involved in pattern separation during learning^39^. This essential cognitive operation ensures efficient encoding of similar experiences or stimuli and their orthogonalization into distinct memory representations. Notably, the DG exhibits sensitivity even to minor differences in input patterns, thereby reducing interference between highly similar memories. This contrasts with recognition memory-related item memory. Item memory is the process of recognizing and remembering specific items or individual pieces of information. It involves storing and retrieving details about a single stimulus, independent of its context or surroundings. Unlike pattern separation, item memory does not focus on differentiation but, instead, on accurately identifying and recalling the characteristics of a particular object or word. While pattern separation involves the transformation of similar representations into distinct, nonoverlapping representations, a complementary cognitive operation called pattern completion refers to the process of completing degraded memory representations by filling in missing information to re-instantiate previously stored representations^40^. Both mechanisms – pattern separation and pattern completion – play a critical role in associative memory, enabling the brain to store memories independently, retrieve memories from partial cues, and flexibly apply stored memories to novel situations^41^.

On the neuronal level, one of the most intriguing features of the DG is adult neurogenesis, a unique phenomenon within the brain that is defined by the continuous generation of new neurons throughout adulthood^42^. Adult hippocampal neurogenesis is a fundamental process that enables the brain to adapt to new experiences and facilitate learning. Numerous models have been proposed to elucidate the impact of neurogenesis on the structure and function of the DG^43–48^. These models explore the potential mechanisms through which the continuous addition of new neurons influences the overall neural architecture and synaptic connectivity within the DG, and how this, in turn, may shape its functional role in cognitive processes.

Unfortunately, the hippocampus, including the DG, is susceptible to various neurological conditions. Reduced neurogenesis and synaptic plasticity in the DG have been particularly associated with cognitive decline observed in aging and neurodegenerative disorders like Alzheimer’s disease^49,50^. Moreover, the significance of changes in hippocampal pattern separation and neurogenesis has also been implicated in various psychiatric disorders, including depression, anxiety, schizophrenia, autism, and post-traumatic stress disorder^45,51–54^. These disorders often manifest cognitive impairments and mood disturbances that could be attributed, at least in part, to that compromised neurogenesis.

The exact mechanisms leading to hippocampal damage in COVID-19 remain a subject of ongoing research. Several pathophysiological mechanisms have been proposed, with neuroinflammation being a significant factor^55^. Being highly susceptible to neuroinflammation, the hippocampus appears to be particularly affected by the cytokine storm induced by COVID-19^56^, a phenomenon characterized by the excessive release of pro-inflammatory cytokines^57^. A recent study demonstrated impaired microglial reactivity in both mice and humans. In mice, alterations in hippocampal neurogenesis and reductions in oligodendrocytes and axon myelin were observed, accompanied by elevated levels of cerebrospinal fluid (CSF) cytokines, particularly eotaxin-1 (CCL11), which were associated with cognitive symptoms in COVID-19 patients^58^. Furthermore, a study with patients diagnosed with post-COVID-19 syndrome reported significant associations between hippocampal volume atrophy three months post-infection and systemic inflammatory marker alterations during the acute stage of COVID-19^32^. Moreover, several other potential contributors have been proposed, including direct viral invasion, immune-mediated responses, vascular dysfunction and hypoxic injury^59^. Importantly, it is again the hippocampus that is particularly susceptible to hypoxia and hypoperfusion, exhibiting greater alterations compared with other brain regions, especially in the Cornu Ammonis and DG subfields^60,61^.

Elevated levels of neuroinflammatory factors have been shown to exert detrimental effects on neurogenesis, hindering the proliferation and differentiation of neural stem cells and ultimately impeding the generation of new neurons^62^. This dovetails with the finding that SARS-CoV-2 infection not only leads to cytokine elevation in the hippocampus but is also accompanied by indicators of increased apoptosis and decreased neurogenesis in the DG^63^. In line with this, Soung et al.^56^ reported a reduced number of neuroblasts in the hippocampal subgranular zone, indicating that fewer neurons are generated. This critical impact underscores the vulnerability of adult hippocampal neurogenesis to the virus and raises the possibility that effects of SARS-CoV-2 infection could also come to expression on the cognitive level. In particular, detrimental effects on hippocampal pattern separation are conceivable as disruptions in neurogenesis potentially lead to alterations in the connectivity and plasticity of hippocampal circuits involved in this cognitive function^64^. The consequences of reduced neurogenesis might result in less distinct neural representations, making it challenging for the brain to accurately separate similar memories or events. Such impaired mnemonic pattern separation could have significant cognitive implications, with individuals recovering from COVID-19 experiencing difficulties in distinguishing between similar experiences or recalling specific details from memory. This, in turn, could lead to memory confusions, decreased precision in learning and recall, and increased susceptibility to memory interference. As mentioned above, pattern separation deficits have been observed in various neuropsychiatric disorders that have been related to neuroinflammation, including post-traumatic stress disorder (PTSD), anxiety disorders, and depression^45,51,52,54^. As these conditions have widely been observed as comorbidities of COVID-19, the impact of COVID-19 can be assumed to create a downward spiral, where virus-induced neuroinflammation, stress, depression, and emotional and psychological trauma collectively impair the neurogenic potential of neural stem cells in the hippocampus. Virus-induced neuroinflammation alone can hinder neurogenesis, but the added effects of stress, (premorbid) depression, and trauma may exacerbate this impairment. Since hippocampal neurogenesis is, in turn, crucial for cognitive and emotional well-being, the reduced neurogenesis observed in COVID-19 survivors^65^ likely further exacerbates both cognitive impairments and mood alterations reported in some individuals following infection^66^. Thus, comprehensive scientific investigation into the abnormal regulation of hippocampal neurogenesis related to cognitive impairment in COVID-19 is urgently needed. Moreover, understanding the potential consequences of COVID-19 on hippocampal neurogenesis and its effects on pattern separation is crucial for developing targeted interventions and cognitive rehabilitation strategies to support individuals affected by the virus and its impact on hippocampal function.

In this study, we aimed to assess the effects of SARS-CoV-2 infection on mnemonic pattern separation in a large-scale online cohort. Although our study does not directly measure neurogenesis or hippocampal function, our hypotheses were informed by prior research linking hippocampal neurogenesis to pattern separation and the potential vulnerability of the dentate gyrus to neuroinflammation caused by COVID-19. Specifically, we compared two groups: participants who reported to have experienced at least one SARS-CoV-2 infection and those who have never tested positive. Based on the theoretical framework linking neuroinflammation to impaired hippocampal neurogenesis, we expected that individuals who have previously been infected show significant deficits specifically in mnemonic pattern separation, while other mnemonic functions, including item recognition memory, are likely to remain intact when compared with participants who have never been infected. Both mnemonic pattern separation and item recognition memory can be assessed behaviorally using the mnemonic similarity task^67^, in which participants are presented with old items that have appeared in the study phase (targets), similar items (lures) that are similar but not identical to the items in the study phase, and new items (foils). We hypothesized that after SARS-CoV-2 infection, participants would not be able to mnemonically separate old from similar items, that is, to identify the lure items as similar but not identical, as expressed in the lure discrimination index (LDI). In contrast, their ability to recognize old items as old should remain unaffected (recognition index, REC). These predictions reflect an indirect but theoretically grounded approach to examining potential disruptions in hippocampus-dependent processes, possibly resulting from COVID-19-related neuroinflammation.

This study also aimed to determine whether the observed cognitive symptoms could be attributed to long-term effects of COVID-19 itself or secondary to neuropsychiatric conditions such as anxiety or depression. By incorporating measures of mental health as covariates, we sought to isolate specific cognitive impairments related to hippocampus-dependent processes from generalized cognitive symptoms driven by neuropsychiatric conditions. While causality cannot be directly established, our approach ensures that observed deficits are not confounded by broader neuropsychiatric symptoms, providing a more precise understanding of the cognitive impacts of COVID-19.

Although the primary aim of this study was to investigate hippocampus-dependent cognitive processes, particularly mnemonic discrimination, as measured by the LDI, we complemented our test battery by tests of executive functions and tonic alertness. While deficits in executive functions, such as attentional control and working memory, could potentially influence performance on hippocampus-related tasks, the inclusion of executive function and working memory assessments was intended to detect a potential selectivity of hippocampus-related impairments and to provide a more comprehensive picture of COVID-19-related cognitive symptoms.

Understanding the broader cognitive profile of COVID-19 survivors is particularly relevant given the evidence for executive dysfunction in the acute stage or within the first three months post-infection, predominantly in individuals of over 50 years and/or with moderate to severe symptoms (e.g.,^27,68–71^), but also in younger participants (see e.g.,^72,73^). Executive dysfunction has been associated with frontoparietal hypometabolism in subacute-stage COVID-19 patients^15,69^ but did not necessarily persist in the long term^69^ (but see^14,74^, and^75^).

Due to inconsistent findings on COVID-19-related executive dysfunction that might be caused by the application of a great diversity of tests and a potential selectivity regarding the specifically affected executive functions, our test battery covered multiple tests of basic executive-function domains, that is, inhibition (go/no-go task, stop-signal task), flexibility (task-switching task), and working memory (n-back task, Corsi block-tapping task).

Beside executive dysfunction, attention-related problems have sometimes also been reported shortly after infection (e.g.,^70,74–77^), and at least partly in the long term^75^, even in participants not reporting any cognitive symptoms^78^. However, in other studies, attention has often been spared, both in the subacute (e.g.,^69,72^) and chronic stage^14^. To compare intrinsic arousal as a stimulus-independent attention measure between previously infected participants and virus-naïve controls, we included an assessment of tonic alertness.

## Results

### Questionnaires

The data of the four questionnaires administered in the survey prior to the cognitive tasks, Patient Health Questionnaire (PHQ-9^79^), Perceived Stress Questionnaire (PSQ-20^80^), General Anxiety Disorder Scale (GAD-7^81^), and WHO well-being index (WHO-5^82^; see also^83^), were analyzed using multiple linear regression to test the effect of infection status on depressiveness, perceived stress, general anxiety, and well-being, respectively, with age, gender, and level of education added as covariates of no interest (see Methods section for sample description and analyses). The analyses revealed a significant difference between individuals with and without a SARS-CoV-2 infection history for depressiveness (PHQ-9), perceived stress (PSQ-20), and well-being (WHO-5), and a marginally significant effect for anxiety (GAD-7; see Table 1). Notably, both infection groups reached depressiveness levels categorized as “mild depression status” (PHQ-9 score range between 5 and 9), This is remarkable, considering that a PHQ-9 score of 7 (on average, 6.82 in the total sample reported here) corresponds to the 88.1 percentile in a healthy norm sample^84^. Importantly, these norming data have been assessed prior to the COVID-19 pandemic, by which people were affected not only through an infection as such but also by the fear of being infected or of losing relatives, and by protection measures such as lockdowns and other social restrictions. An alternative explanation for the higher depressiveness scores could also be a general increase in depression prevalence in recent years, irrespective of a pandemic^85^.

In a similar vein, both participant groups reached an anxiety symptom level categorized as “mild anxiety” (GAD-7 score range between 5 and 9) and they both fall below the WHO-5 well-being cut-off score of 50, which is considered an indicator of poor emotional state that requires further testing^83^.

**Table 1:** Mean scores of the questionnaires (standard deviations in parentheses) and effects of infection status on the respective scores, gained by the corresponding multiple regression analyses.

Questionnaire	Infection status^a^			*b*	β	*t* (df)	*p*
	Previously not infected	Previously infected	Total				
Depressiveness (PHQ-9)	6.14 (4.41)	7.23 (5.55)	6.82 (5.18)	1.06	.10	3.52 (1225)	.002**
Perceived Stress (PSQ-20)	39.93 (21.22)	43.12 (21.34)	41.90 (21.34)	3.34	.08	2.71 (1228)	.021*
General Anxiety (GAD-7)	5.65 (4.15)	6.15 (4.32)	5.96 (4.26)	0.45	.05	1.79 (1200)	.073^†^
Well-being (WHO-5)	48.34 (24.61)	44.62 (24.31)	46.02 (24.48)	-3.53	− .07	-2.45 (1214)	.029*

Test ranges are as follows: PHQ-9: 0–27, PSQ-20: 0–100, GAD-7: 0–21, WHO-5: 0–100. Higher scores indicate more severe depressiveness (PHQ-9), more perceived stress levels (PSQ-20), more severe anxiety (GAD-7) and a better overall well-being (WHO-5). Please note that the PSQ-20 was assessed as an adapted version, that is, referring to the last two weeks instead of four weeks as in the original version. Outliers were removed according to Tukey^86^(> 1.5 interquartile ranges below the first or above the third quartile). Age, gender, and level of education were added to the regression model as covariates of no interest.

^a^ Previously uninfected = 0, previously infected = 1.

^†^*p* < .10. *p < .05. **p < .01. *p* values are Bonferroni-Holm corrected to adjust for multiple comparisons.

### Long-term memory

#### Mnemonic similarity task (MST)

As in the analyses of all cognitive tasks, we used multiple linear regression to determine the effect of infection on the dependent variables of interest, controlling for the covariates age, gender, level of education, depressiveness, anxiety, and stress (see Methods section for further detail). In the overall regression model predicting the LDI (*P*(“similar” response | lure) – *P*(“similar” response | foil)) as a measure of mnemonic pattern separation, infection status was predictive for the LDI, controlling for the covariates specified above (see Table 2, Model 1). In particular, the analysis revealed a diminished LDI for participants with prior SARS-CoV-2 infection, compared with participants without an infection history (previously infected: *M* = .24, *SD* = .17; previously not infected: *M* = .32, *SD* = .19; see Table 2, Model 1). Consistent with prior research^87,88^, advancing age was also linked to a reduced LDI independent of the other regressors (see Table 2, Model 1). A further exploratory regression analysis including the Infection status x Age interaction in addition to the previous model confirmed the effect of infection status on the LDI, beyond the other regressors (Table 2, Model 2). However, the main effect of age did not reach significance (BF_10_ = 0.41), indicating anecdotal evidence for the absence of an additional age effect. Likewise, the Infection status x Age interaction did not emerge as meaningful (BF_10_ = 1.18), indicating that there was no conclusive evidence that the infection-related LDI impairment depended on participant age.

To investigate potential changes in the infection-associated decrease in the LDI over time post-infection, we conducted a subgroup analysis exclusively involving participants who had tested positive in the past. We introduced the time elapsed between the positive PCR test and study participation as an additional fixed factor to predict the LDI, while controlling for age, gender, level of education, depressiveness, anxiety, and stress. Notably, the time interval between the positive PCR test and study participation significantly predicted the LDI, but revealing a *lower* LDI when more time has passed since the positive PCR test (see Table 2, Model 3a). Strikingly, a subsample of previously infected participants self-reporting to have fully recovered from any acute or post-acute symptoms and to be able to pursue their daily activities still exhibited a significantly reduced LDI, compared with individuals without an infection history (see Table 2, Model 4).

To assess the impact of the quantity of post-acute and long COVID symptoms on the LDI in participants who had tested positive in the past, we added a composite infection sequelae factor to the regression model. This factor encapsulated the total number of persisting post-acute and long COVID symptoms encompassing tiredness, apathy, dyspnea, anosmia or ageusia, headache and limb pain, muscle- and limb-related symptoms, pulmonary dysfunction, heart problems, rhinitis, gastrointestinal symptoms, vertigo, vomiting, susceptibility to inflammation, severe coughing, sore throat, eczema, swollen lymph nodes, conjunctivitis, memory impairment, wordfinding problems, and other prolonged COVID or long COVID symptoms. Note that anosmia and ageusia as well as tiredness were assessed within two survey items: once as persisting post-acute symptom and once as long COVID symptom. If a participant reported anosmia and ageusia or tiredness at least once (i.e., either as persisting or long COVID symptom), we counted this response as one symptom in all cognitive task analyses, regardless of whether a participant reported this symptom as persisting only, long COVID only, or both persisting and long COVID. The number of infection sequelae was predictive for the LDI, whilst controlling for the influence of age, gender, education level, depressiveness, anxiety, and stress (see Table 2, Model 3b). A higher number of infection sequelae was associated with a lower LDI.

For the REC index (*P*(“old” response | target) – *P*(“old” response | foil)) as a measure of item recognition, the overall regression model encompassing infection status, age, gender, level of education, depressiveness, anxiety, and stress yielded no clear evidence in favor of an effect of either infection status or age (infection status: BF_10_ = 0.86, previously infected: *M* = .80, *SD* = .10, previously not infected: *M* = .81, *SD* = .09; age: BF_10_ = 0.38; see Table 3, Model 5). In an additional model including selectively only participants who had previously tested positive, the time elapsed since the PCR test did not predict the REC index (BF_10_ = 0.74), whilst controlling for the specified covariates (see Table 3, Model 6a). Assessing the impact of the number of post-acute sequelae on the REC index in the same subsample did not reveal an effect on the REC index (BF_10_ = 0.98; see Table 3, Model 6b), indicating that the data were virtually equivocal regarding a potential influence of symptom count on item recognition performance.

In addition to the primary indices, LDI and REC, we computed the pattern completion (PC) bias (P(“old” response | lure) – P(“similar” response | lure)) to investigate whether participants who had previously been infected demonstrated a tendency to incorrectly endorse similar lures as old items. Infection status significantly predicted the PC bias, whilst controlling for the specified covariates (see Table 4, Model 7). Furthermore, an age effect was observed, mirroring the LDI results. Adding the time between the positive PCR test and study participation to the model in the subgroup of participants who had tested positive did not reveal time as a predictive factor (BF_10_ = 0.83; see Table 4, Model 8a). Similarly, the number of sequelae did not predict the PC bias, whilst controlling for the specified covariates (BF_10_ = 0.84 ; see Table 4, Model 8b). In both cases, BF_10_ were around 0.8, indicating only anecdotal evidence for the null – that is, no robust evidence that time since infection or symptom count affected the pattern completion bias beyond the main effects already captured.

**Table 2:** Linear regression models to predict the LDI.

Variable	Whole sample						Subsample: previously infected participants						Subsample: recovered and previously not infected participants		
	Model 1			Model 2			Model 3a			Model 3b			Model 4		
	*b* (SE)	β	*t* (*p*)	*b* (SE)	β	*t* (*p*)	*b* (SE)	β	*t* (*p*)	*b* (SE)	β	*t* (*p*)	*b* (SE)	β	*t* (*p*)
Intercept	.26 (.04)		6.58 (< .001***)	.26 (.04)		6.69 (< .001***)	.25 (.06)		4.36 (< .001***)	.19 (.05)		4.03 (< .001***)	.27 (.04)		6.45 (< .001***)
Infection status^a^	− .08 (.03)	− .21	-2.47 (.015*)	− .08 (.03)	− .23	-2.70 (.008**)							− .07 (0.03)	− .18	-2.12 (.036*)
Age	-<.01 (< .01)	− .18	-2.05 (.042*)	-<.01 (< .01)	− .04	-0.29 (.776)	-<.01 (< .01)	− .28	-2.29 (.025*)	-<.01 (< .01)	− .29	-2.41 (.019*)	-<.01 (< .01)	− .17	-1.84 (.068^†^)
Gender^b^	< .01 (.03)	< .01	0.04 (.967)	.01 (.03)	.02	0.25 (.805)	.02 (.04)	.05	0.44 (.665)	< .01 (.04)	0.01	0.09 (.932)	− .01 (.03)	− .04	-0.41 (.682)
Level of education^c^	.04 (.03)	.12	1.42 (.158)	.04 (.03)	.10	1.24 (.218)	.06 (.04)	.18	1.61 (.112)	.04 (.04)	.13	1.10 (.273)	.03 (.03)	.09	1.01 (.313)
Depressiveness	.01 (.01)	.21	1.50 (.137)	.01 (.01)	.19	1.41 (.160)	< .01 (.01)	.02	0.11 (.912)	.01 (.01)	.17	0.89 (.377)	.01 (.01)	.15	1.06 (.291)
Anxiety	− .01 (.01)	− .16	-1.01 (.315)	− .01 (.01)	− .18	-1.15 (.253)	-<.01 (.01)	− .05	-0.24 (.812)	− .01 (.01)	− .20	-0.93 (.355)	− .01 (.01)	− .14	-0.86 (.389)
Stress	< .01 (< .01)	.07	0.47 (.639)	< .01 (< .01)	.10	.63 (.533)	< .01 (< .01)	.07	0.31 (.760)	< .01 (< .01)	− .26	1.11 (.271)	< .01 (< .01)	.10	0.63 (.530)
Age x Infection status				-<.01 (< .01)	− .20	-1.60 (.112)									
Months since PCR test							− .01 (< .01)	− .23	-2.06 (.044*)						
Number of sequelae										− .02 (0.1)	− .30	-2.13 (.037*)			
*R*²	.14			.15			.23			.24			.12		
Adjusted *R*²	.09			.10			.16			.16			.06		
*F* (*p*)	2.94 (.007**)			2.93 (.005**)			.97 (0.009**)			3.02 (.008**)			2.15 (.043*)		

The LDI was calculated as *P*(“similar” | lure) - *P*(“similar” | foil). All *p* values are reported twotailed.

^a^Previously uninfected = 0, previously infected = 1.

^b^Female = 0, male = 1.

^c^Without post-secondary education = 0, with post-secondary education = 1.

^†^*p* < .10. *p < .05. **p < .01. ***p < .001.

**Table 3:** Linear regression models to predict the REC index.

Variable	Whole sample			Subsample: previously infected participants					
	Model 5			Model 6a			Model 6b		
	b (SE)	β	t (*p*)	b (SE)	β	t (*p*)	b (SE)	β	t (*p*)
Intercept	.76 (.02)		36.08 (< .001***)	.71 (.03)		20.70 (< .001***)	.73 (.03)		25.89 (< .001***)
Infection status^a^	− .02 (.02)	− .11	-1.37 (.172)						
Age	-<.01 (< .01)	-<.01	-0.05 (.963)	< .01 (< .01)	.06	0.53 (.597)	< .01 (< .01)	.06	0.53 (.595)
Gender^b^	.07 (.02)	.31	3.66 (< .001***)	.09 (.02)	.40	3.56 (< .001***)	.09 (.02)	.43	3.77 (< .001***)
Level of education^c^	< .01 (.02)	.03	0.33 (.740)	− .01 (.02)	− .06	-0.51 (.610)	-<.01 (.02)	− .02	-0.20 (.843)
Depressiveness	-<.01 (< .01)	− .07	-0.54 (.589)	-<.01 (< .01)	-<.01	-0.02 (.985)	-<.01 (< .01)	− .10	-0.53 (.599)
Anxiety	-<.01 (< .01)	− .21	-1.37 (.174)	− .01 (< .01)	− .29	-1.45 (.153)	-<.01 (.01)	− .19	-0.92 (.363)
Stress	< .01 (< .01)	.39	2.52 (.013*)	< .01 (< .01)	.41	1.87 (.065^†^)	< .01 (< .01)	.28	1.23 (.225)
Months since PCR test				< .01 (< .01)	.13	1.31 (.262)			
Number of sequelae							.01 (.01)	.20	1.41 (.163)
*R*²	.14			.23			.13		
Adjusted *R*²	.10			.15			.15		
*F* (*p*)	3.06 (.005**)			2.83 (.012*)			2.96 (.009**)		

The REC index was calculated as *P*(“old” | target) - *P*(“old” | foil). All *p* values are reported twotailed.

^a^Previously uninfected = 0, previously infected = 1. ^b^Female = 0, male = 1. ^c^Without post-secondary education = 0, with post-secondary education = 1.

^†^*p* < .10. *p < .05.**p < .01. ***p < .001.

**Table 4:** Linear regression models to predict the PC bias.

	Whole sample			Subsample: previously infected participants					
Variable	Model 7			Model 8a			Model 8b		
	b (SE)	β	t (*p*)	b (SE)	β	t (*p*)	b (SE)	β	t (*p*)
Intercept	.03 (.07)		0.53 (.599)	.07 (.10)		0.66 (.511)	.14 (.09)		1.57 (.121)
Infection status^a^	.13 (.05)	.21	2.50 (.014*)						
Age	.01 (< .01)	.29	3.42 (< .001***)	.01 (< .01)	.39	3.25 (.002**)	.01 (< .01)	.40	3.36 (.001**)
Gender^b^	.01 (.06)	.02	0.22 (.825)	.01 (.07)	.02	0.18 (.856)	.03 (.07)	.04	0.39 (.700)
Level of education^c^	− .05 (.05)	− .07	-0.87 (.386)	− .06 (.07)	− .09	-0.79 (.433)	− .04 (.07)	− .06	-0.49 (.627)
Depressiveness	− .01 (.01)	− .13	-0.92 (.362)	< .01 (.01)	.01	0.06 (.954)	− .01 (.01)	− .08	-0.41 (.684)
Anxiety	< .01 (.01)	.04	0.28 (.781)	-<.01 (.02)	− .04	-0.19 (.850)	< .01 (.02)	.05	0.24 (.815)
Stress	< .01 (< .01)	.02	0.12 (.905)	< .01 (< .01)	.05	0.23 (.819)	-<.01 (< .01)	− .06	-0.27 (.787)
Months since PCR test				.01 (.01)	.14	1.25 (.216)			
Number of sequelae							.02 (.02)	.18	1.26 (.214)
*R*²	.15			.23			.23		
Adjusted *R*²	.11			.15			.15		
*F* (*p*)	3.36 (.003**)			2.86 (.011*)			.86 (.011*)		

The PC bias was calculated as *P*(“old” | lure) - *P*(“similar” | lure). All *p* values are reported two-tailed.

^a^ Previously uninfected = 0, previously infected = 1. ^b^ Female = 0, male = 1. ^c^ Without post-secondary education = 0, with post-secondary education = 1.

*p < .05. **p < .01. ***p < .001.

To rule out that the LDI reduction in the group with an infection history is driven by a speed-accuracy trade-off, we further tested if the correct “similar” responses by the previously infected individuals were faster than those by the previously uninfected participants. This was not the case (previously infected:* M* = 1.36 s, *SD* = 0.15 s, previously not infected:* M* = 1.36 s, *SD* = 0.14 s; *t*(136) = -0.08, *p* = .938, BF_10_ = 0.18). The BF_10_ indicates moderate evidence in favor of the null hypothesis, supporting the notion that there was no difference in response speed between the groups. Following the same rationale, we compared between-group response latencies for correct “old” responses, to rule out that a potential REC impairment by individuals with an infection history might have been covered as they took more time to correctly respond “old”. Again, there was no significant difference between infection status groups (previously infected: *M* = 1.06 s; *SD* = 0.13 s, previously not infected: *M* = 1.08 s, *SD* = 0.13 s; *t*(136) = 0.81, *p* = .420, BF_10_ = 0.25). This BF_10_ suggests weak (anecdotal) evidence for the null hypothesis—that is, the data provide no clear indication of a meaningful latency difference for item recognition responses either.

### Alertness

The linear regression model to test whether infection status serves as a predictor for mean response latencies in the alertness task (see Methods section for further detail), controlling for the specified covariates, did not explain a significant proportion of variability in mean response latencies (see Supplementary Information, Table S1). Specifically, infection status failed to predict response latencies, *t* = 1.43, *p* = .156, BF_10_ = 0.45 (previously infected: *M* = 359 ms, *SD* = 44 ms, previously not infected: *M* = 351 ms, *SD* = 30 ms). The BF_10_ value indicates only anecdotal evidence for the null, suggesting that the groups may not differ substantially in their mean reaction time.

Analogously to response-time analyses, a linear regression model was deployed to assess the potential predictive role of infection status in alertness task accuracy. The model, including infection status and the specified covariates, failed to account for a significant proportion of variability in mean accuracy. Infection status did not demonstrate a significant predictive value, *t* < 1, BF_10_ = 0.18 (see Supplementary Information, Table S1; previously infected: *M* = 99.29% correct, *SD* = 1.28%, previously not infected: *M* = 99.30% correct, *SD* = 1.45%). The BF_10_ factor provides moderate evidence for the null hypothesis, suggesting that previously infected and non-infected individuals performed with comparable accuracy.

### Executive functions

#### Working memory

##### Corsi block tapping

Infection status significantly predicted forward block span (see Methods section for further detail) after accounting for age, gender, level of education, depressiveness, anxiety, and stress, *b* = -0.31, *SE* = 0.15, β = − 0.12, *t* = -2.01, *p* = .045 (see Supplementary Information, Table S2). Participants without a previous SARS-CoV-2 infection demonstrated a greater ability to repeat longer sequences compared to those who had tested positive in the past (*M* = 5.39 blocks, *SD* = 1.02 blocks, *M* = 5.74 blocks, *SD* = 1.31 blocks). In contrast, when applying the same regression model to the backward version of the Corsi block-tapping task, infection status did not significantly predict backward block span, *t* = -1.44, *p* = .151. The Bayes factor was BF_10_ = 0.85 indicating only weak (anecdotal) evidence favoring no infection effect on the backward span, even though the descriptive pattern was similar to the forward version (previously infected: *M* = 5.43 blocks, *SD* = 0.98 blocks, previously not infected: *M* = 5.71 blocks, *SD* = 1.37 blocks). In other words, infected and non-infected groups performed comparably on the more difficult backward span task, despite a small group difference in the easier forward span task.

##### N-back task

In the 2-back working memory task, infection status was not a meaningful predictor of performance. For the sensitivity measure *d*’ (see Methods section), we found *t* < 1 and BF_10_ = 0.26, indicating moderate evidence for no difference in working memory accuracy between previously infected and non-infected participants (previously infected: *M* = 2.21, *SD* = 0.80, previously not infected: *M* = 2.27, *SD* = 0.69; see Supplementary Information, Table S3). Similarly, infection status had no significant effects on response latencies in the n-back, *t* = -1.11, *p* = .270, BF_10_ = 0.49, controlling for age, gender, level of education, depressiveness, anxiety, and stress (see Supplementary Information, Table S3). This BF_10_ suggests only anecdotal evidence for the null, meaning the data did not provide a clear indication of any slowing or speeding of responses due to infection status. Taken together, performance on the n-back task was very similar for the two groups, with no reliable evidence of an infection-related deficit in this working memory measure.

#### Flexibility

The regression model predicting *specific switch costs in response latencies* by infection status, age, gender, education, depressiveness, anxiety, and stress, did not account for a significant proportion of variance in specific switch costs (see Supplementary Information, Table S4; see Methods section for further detail on the task and analyses). Similarly, the predictive value of infection status also lacked significance, *t* < 1, BF_10_ = 0.35 (previously infected: *M* = 295 ms, *SD* = 189 ms, previously not infected: *M* = 290 ms, *SD* = 179 ms), controlling for the other regressors. This BF_10_ provides evidence leaning toward the absence of any infection effect on switch costs (approximately moderate evidence for the null). When applying the same model to predict the *mixing costs in response latencies*, infection status did again not emerge as a significant predictor for response-latency mixing costs, *t* < 1, BF_10_ = 0.41 (see Supplementary Information, Table S4; previously infected: *M* = 326 ms, *SD* = 245 ms, previously not infected: *M* = 316 ms, *SD* = 210 ms). The BF_10_ of 0.41 indicates only anecdotal evidence for no effect, so we cannot conclusively assert the null in this case. In general, response times for correct responses did not differ markedly between groups, neither in switch trials in task-heterogeneous blocks (previously infected: *M* = 1322 ms, *SD* = 429 ms, previously not infected: *M* = 1268 ms, *SD* = 335 ms), *t*(323) = -1.14, *p* = .254, BF_10_ = 0.24, nor in non-switch trials in task-heterogeneous blocks (previously infected: *M* = 1007 ms, *SD* = 362 ms, previously not infected: *M* = 960 ms, *SD* = 292 ms), *t*(323) = -1.16, *p* = .245, BF_10_ = 0.25, nor in (non-switch) trials in task-homogeneous blocks (previously infected: *M* = 750 ms, *SD* = 202 ms, previously not infected: *M* = 714 ms, *SD* = 145 ms), *t*(323) = -1.68, *p* = .094, BF_10_ = 0.49. In sum, all BF_10_ for the task-switching response-time measures were below 0.5, indicating that the data provided at most modest evidence of no difference in switching performance between groups. There was no indication of any substantial slowing in task-switching ability due to infection.

Analogously to response-time analyses, the regression model predicting *specific switch costs in error rates* by infection status, age, gender, education, depressiveness, anxiety, and stress, did not explain a significant proportion of variance in specific switch costs (see Supplementary Information, Table S4). This was also the case for the predictive power of infection status on specific error-rate switch costs, *t* < 1, BF_10_ = 0.34 (previously infected: *M* = .02, *SD* = .05, previously not infected: *M* = .03, *SD* = .05), controlling for the other regressors. While the model to assess *mixing costs in error rates* reached marginal significance (see Supplementary Information, Table S4), infection status did not contribute significantly to the prediction of mixing costs, *t* < 1, BF_10_ = 0 .34 (previously infected: *M* = .03, *SD* = .06, previously not infected: *M* = .03, *SD* = .06). Thus, whether looking at reaction times or error rates, we found no evidence that prior infection adversely affected task-switching performance. These findings underscore the limited impact of infection status on flexibility within the context of specific and mixing costs in both response latencies and error rates.

#### Inhibition

##### Go/no-go task

With participant added as random intercept, infection status was not predictive for accuracy as measured by *d’* (see Methods section), *t* < 1, BF_10_ = 0.21 (see Supplementary Information, Table S5; previously infected: *M* = 2.83, *SD* = 0.83, previously not infected: *M* = 2.84, *SD* = 0.84), after controlling for age, gender, level of education, depressiveness, anxiety, stress, and the within-participants factor task phase (first, second). The BF_10_ of 0.21 suggests moderate evidence in favor of the absence of a group difference in inhibitory control performance. Similarly, there was no effect of infection status on false alarm rate (i.e., commission errors), *t* < 1, BF_10_ = 0.24 (see Supplementary Information, Table S5; previously infected: *M* = .29, *SD* = .18, previously not infected: *M* = .30, *SD* = .20), again indicating moderate evidence for no difference between groups.

Response latencies were not predicted by infection status, *t* < 1, BF_10_ = 0.23 (see Supplementary Information, Table S5). Instead, the within-subjects factor trial type (go trials, no-go trials), significantly predicted response latencies beyond the other regressors, *b* = -0.06, *SE* = < 0.01, β = − .44, *t* = -25.46, *p* < .001, revealing that (correct) go-trial responses were slower than (incorrect) no-go-trial responses in both infection groups (go trials- previously infected: *M* = 394 ms, *SD* = 61 ms, go trials - previously not infected: *M* = 398 ms, *SD* = 58 ms; no-go trials - previously infected: *M* = 333 ms, *SD* = 67 ms, no-go trials - previously not infected *M* = 336 ms, *SD* = 57 ms). Consistent with these near-identical means, the comparison of overall response times yielded *t* < 1, BF_10_ = 0.14, providing fairly strong evidence that there was no systematic group difference in response speed on the go/no-go task.

##### Stop-signal task

Whilst controlling for age, gender, level of education, depressiveness, anxiety, and stress, infection status was not predictive for stop-signal reaction time (SSRT; see Methods section), *t* < 1, BF_10_ = 0.15 (see Supplementary Information, Table S6; previously infected: *M* = 586 ms, *SD* = 117 ms, previously not infected: *M* = 587 ms, *SD* = 115 ms). A BF_10_ around 0.15 indicates moderate evidence favoring the null hypothesis — in this case, that previously infected and non-infected participants had comparable inhibitory control speed. Consistently, the groups’ overall reaction times on go trials in the stop-signal task were virtually identical (previously infected: *M* = 613 ms, *SD* = 13 ms, previously not infected: *M* = 611 ms, *SD* = 13 ms, *t* < 1, BF_10_ = 0.15). Thus, both the primary measure of inhibition (SSRT) and general response speed provided convergent evidence that SARS-CoV-2 infection status did not noticeably impact performance on the stop-signal task.

### Additional analyses

Beside the LDI in the MST, the only measure within our comprehensive test battery that revealed a detrimental effect of infection status on cognitive functioning was the Corsi block-tapping forward task. Given the established connection between hippocampal structures and visuo-spatial working memory in the Corsi block-tapping task^89^, we sought to explore whether an individual’s block span in this task could be linked to their LDI in the MST. The underlying hypothesis was that impaired performance in the Corsi task might be attributed to infection-induced hippocampal malfunctioning. To further address this question, we merged the Corsi forward dataset with the MST dataset (*N* = 87, previously infected:* n* = 59, previously not infected: *n* = 28). This revealed a positive correlation of the forward block span and the LDI, *r* = .29, *t*(85) = 2.76, *p* = .007. Additionally, there was a negative correlation between the forward span and the PC bias that approached significance, *r* = − .18, *t*(85) = -1.67, *p* = .098, suggesting a trend whereby better Corsi performance might relate to a lower tendency to false alarm on similar lures. There was no indication of a relationship between forward block span and the REC index, *t* < 1, BF_10_ = 0.27, with the BF_10_ providing moderate evidence for the absence of any correlation between these measures. A parallel pattern emerged for the backward block span, showing a positive correlation with the LDI, *r* = .23, *t*(82) = 2.17, *p* = .033, and no correlation with the REC index, *t* < 1, BF_10_ = 0.36, again suggesting only weak evidence of any association. In the case of the backward Corsi task, the PC bias did not exhibit a significant correlation with block span, *r* = − .16, *t*(82) = -1.46, *p* = .149, BF_10_ = 0.89. A BF_10_ close to 1 indicates anecdotal evidence at best, so we cannot draw a firm conclusion about the presence or absence of a backward span–PC bias relationship. In sum, the observed correlations between block span and MST measures suggest a potential link between infection-induced hippocampal dysfunction and impaired performance in visuo-spatial working memory tasks, although the evidence is stronger for some relationships (LDI) than for others (REC).

We conducted a further exploratory analysis to evaluate a potential effect of a vaccination on the LDI. The question if participants had received a COVID-19 vaccine prior to infection (yes/no) was added to the survey in November 2021 and was not mandatory. Of the participants included in the MST analyses, 24 indicated to have been vaccinated prior to infection, 37 had not been vaccinated, and 15 did not provide a response or have participated before the question had been added to the survey. We included the vaccination variable in a model predicting the LDI in a subsample of *n* = 61 previously infected participants, including age, gender, level of education, depressiveness, anxiety, and stress as predictors of no interest. This analysis did not provide evidence of a vaccination effect on the LDI, *b* = .02, *SE* = .05, β = .04, *t* < 1, BF_10_ = 0.49, indicating that the data were largely inconclusive regarding any vaccination benefit or detriment – essentially, LDI performance was similar whether or not participants had been vaccinated prior to infection.

## Discussion

In this study, our goal was to thoroughly investigate cognitive impairment related to COVID-19. We collected data from a large online cohort of over 1,400 participants, comprising both individuals with a history of infection and those who had never tested positive. We employed experimental cognitive tasks that provide objective behavioral measures of performance, such as reaction times and accuracy, rather than relying solely on subjective self-reports. Although the tasks were self-administered in an online format, they were standardized and designed to yield quantifiable, reproducible outcomes, ensuring objectivity in an experimental sense. The key findings are as follows: Participants who had tested positive for SARS-CoV-2 in the past exhibited higher levels of depressiveness, perceived stress, and anxiety compared with those who had never been infected. They also reported lower levels of well-being. In terms of long-term memory, a diminished LDI was observed in participants with prior SARS-CoV-2 infection, suggesting a potential negative impact on hippocampus-dependent pattern separation. Importantly, this negative effect of infection status on the LDI was observed after correcting for effects of age, gender, level of education, depressiveness, anxiety, and stress. In line with previous literature^87,88^, a significant age effect indicated an LDI reduction with increasing age, in the absence of an Infection Status x Age interaction. Regarding PC, infection status significantly predicted the PC bias, with participants who had tested positive for SARS-CoV-2 showing a higher tendency to incorrectly endorse lures as old items. An age effect was also observed, consistent with the LDI results. In the alertness task, no significant differences were found in mean response latencies or accuracy between participants with and without a SARS-CoV-2 infection history, indicating that tonic alertness was not markedly affected by the infection. Similarly, neither SSRT in the stop-signal task nor accuracy or response times in the go/no-go task significantly differed between the two groups, indicating no notable impact of infection status on response inhibition. In the task-switching task, neither mixing costs nor specific switch costs were affected by infection status, suggesting the absence of a detectable effect of infection status on flexibility. Regarding working memory, we observed a similar pattern for the n-back results, with no significant effect of infection status on accuracy or response latencies. However, participants with a history of SARS-CoV-2 infection showed impaired performance on the Corsi block-tapping forward task, which correlated with their LDI scores. This suggests a potential link between hippocampal dysfunction and visuo-spatial working memory deficits in infected individuals. Notably, the expected deficit in mnemonic pattern separation and the reduced Corsi forward block span were the only measures for which a negative effect of a SARS-CoV-2 infection was observed.

Given the susceptibility of hippocampal neurogenesis to COVID-19 and its potential impact on pattern separation, cognitive consequences related to memory precision were anticipated. Specifically, we expected significant deficits in mnemonic pattern separation among individuals who had tested positive for COVID-19, compared to previously not infected participants, while other mnemonic functions, such as item recognition memory, would largely remain intact. Our results exactly confirm this hypothesis, showing that previously infected individuals exhibited a marked and selective impairment in mnemonic discrimination, which relies on hippocampus-dependent pattern separation, leaving item recognition memory intact. This suggests that compromised hippocampal neurogenesis following SARS-CoV-2 infection may contribute to the memory deficits observed in COVID-19 survivors. However, while the MST is a valuable tool for assessing cognitive functions related to pattern separation, it is important to acknowledge that it serves only as an indirect measure of neurogenesis and the integrity of the DG. To account for cognitive processes beyond hippocampus-dependent pattern separation, we also examined the REC score, which assesses item recognition memory (i.e., the ability to recognize a target item as “old”) that should be less reliant on hippocampal processing and rather reflect the integrity of other memory-related structures of the medial temporal lobe, such as the perirhinal cortex (see^90^ for a model assigning item recognition to perirhinal processing, and contextual, episodic representations to the hippocampus). The absence of significant group differences in REC scores suggests that item memory remains intact in COVID-19 survivors, further supporting the specificity of the observed deficits in hippocampus-dependent processes. This distinction further emphasizes the MST’s utility in isolating hippocampus-dependent processes, specifically pattern separation, from other cognitive functions, such as item recognition memory, which rely on perirhinal cortex integrity^90^. However, it is important to note that the MST’s reliance on behavioral performance to infer hippocampal function means that it cannot directly measure neurogenesis or the structural integrity of specific hippocampal regions. Neurogenesis and DG integrity are complex processes influenced by various factors, and their direct assessment typically requires neuroimaging techniques, such as high-resolution fMRI, or post-mortem histological analysis. Nevertheless, the MST provides valuable insights into hippocampus-dependent cognitive processes, such as pattern separation, through its reliance on well-established behavioral markers. While it cannot directly measure neurogenesis or DG integrity, its sensitivity to functional deficits allows for indirect inferences about hippocampal involvement in cognitive impairments. This indirect approach is particularly useful in large-scale, non-invasive studies, where neuroimaging or histological techniques are not feasible. The MST infers the functional status of the DG through participants’ ability to discriminate between similar but not identical stimuli—a cognitive process believed to be supported by neurogenesis. Moreover, while deficits observed in the MST lure-discrimination performance can suggest impaired neurogenesis, they can also result from other factors affecting the hippocampus, such as overall hippocampal atrophy, which may not be directly related to neurogenesis. Therefore, although the MST can provide valuable insights into cognitive processes associated with hippocampal function, it should be complemented with direct measures of neurogenesis and hippocampal integrity to provide a more comprehensive understanding of the underlying biological mechanisms.

Nevertheless, an indirect line of evidence supporting the notion that behavioral pattern separation may be neurogenesis-dependent comes from studies showing that performance on this task is influenced by several known up-regulators and down-regulators of neurogenesis. Evidence suggests that performance on the MST is influenced by factors known to regulate neurogenesis. For example, Déry et al.^91^ found that participants who improved their fitness through a 6-week aerobic exercise program showed greater improvements on a visual mnemonic pattern separation task. Conversely, individuals with higher scores on the Beck Depression Inventory-II (BDI) demonstrated significant deficits in identifying lures as similar, compared with their less-depressed counterparts, without differences in identifying repetitions or novel objects^91^. Similarly, higher scores on the Depression Anxiety Stress Scale negatively correlated with performance on mnemonic pattern separation tasks^92^. Aging, another factor known to downregulate neurogenesis, also impacts pattern separation performance. High-resolution fMRI and DTI studies have shown that aging is associated with hyperactivity in the DG/CA3 region, degradation of the perforant pathway, and mild cognitive impairment (MCI;^41,93,94^). These studies all link impaired hippocampal processing to decreased performance on pattern separation tasks. Thus, factors such as exercise, stress, and aging, which are known to influence neurogenesis in rodents, similarly correlate with cognitive performance on tasks like the MST in humans. This alignment supports the validity of using the MST task to measure cognitive impacts related to hippocampal neurogenesis. Thus, despite the lack of a direct link between hippocampal neurogenesis and mnemonic pattern separation, together with previous evidence of compromised hippocampal neurogenesis after SARS-CoV-2 infection^95^, our MST results contribute to a more comprehensive understanding of the underlying neuropathophysiological mechanisms. After all, to our knowledge, the pattern of the MST results reported here are the first to show a clearly expressed behavioral post-infection deficit that is selective for mnemonic discrimination.

Our study found that a longer duration between a positive PCR test and study participation was associated with a lower LDI. This finding can be interpreted in the context of several key observations from existing literature. Firstly, it aligns with the understanding that neurogenesis recovery in the DG is a prolonged and gradual process. Kempermann et al.^96^ described adult hippocampal neurogenesis as a tightly regulated process that involves a well-characterized sequence of maturation stages in rodents. Immature neuroblasts, after exiting the cell cycle, go through a series of differentiation phases before becoming fully mature, highlighting the complexity and time required for neurogenesis recovery. Singer et al.^97^ and Yun et al.^98^ found that the recovery of neurogenesis in the DG is both slow and partial, often taking several weeks to months for even a transient reduction to return to normal levels. Furthermore, Kohler et al.^99^ emphasized that the maturation of granule cells in the DG of nonhuman primates takes at least six months, indicating that any disruption in neurogenesis, such as from stress or illness, could have a prolonged impact on hippocampal function and cognitive recovery. Correspondingly, Lemaire et al.^100^ demonstrated that disruption of neurogenesis in the DG impairs learning abilities even months after the initial disruption. In our study, however, we not only found no improvement in symptoms but also a deterioration. It is conceivable that during the long neurogenesis recovery period, the formation of new neurons and their integration into existing neural circuits may be incomplete, possibly leading to continued impairments in pattern separation. However, persistent stress, often associated with prolonged illness or recovery periods, can not only suppress the proliferation of new neurons but also alter the functioning of existing ones. This ongoing stress response may worsen cognitive functions over time, including pattern separation, as the hippocampus becomes increasingly dysregulated. Moreover, patients recovering from illness may experience a cumulative cognitive load due to the prolonged duration of recovery. This includes dealing with stress, physical symptoms, and mental fatigue, all of which can contribute to cognitive decline. Over time, this sustained cognitive load can further impair neurogenesis and hippocampal function, leading to worse pattern separation performance. Prolonged periods of reduced neurogenesis might then lead to degeneration or maladaptive changes in existing neural circuits within the hippocampus. As new neurons fail to mature and integrate properly, the existing circuits may deteriorate due to a lack of reinforcement and plasticity, exacerbating cognitive impairments over time. In some cases, compensatory mechanisms within the brain may initially help cope with reduced neurogenesis, but over time, these mechanisms can become exhausted or maladaptive. As the brain’s ability to compensate diminishes, pattern separation may deteriorate further.

Unlike earlier studies^5,6,13,101,102^, our findings indicate no difference in alertness or in most tasks evaluating executive functions such as updating, inhibition and flexibility (assessed through the n-back task, the stop-signal task, the go/no-go task, and task switching). The discrepancies between our findings and those of previous studies may stem from differences in patient severity. Previous research often involved participants with severe COVID-19 symptoms, whereas our study focused on individuals who were largely non-hospitalized. Hence, this contrast is likely due to the milder symptoms experienced by our participants, coupled with the longer duration since infection. These factors suggest that some functional deficits might not be apparent in individuals with milder COVID-19 cases to a noticeable degree. Beside the influence of illness severity, the specifications of the tasks used for cognitive assessment also widely varies in the literature. However, participants with a history of SARS-CoV-2 infection demonstrated impaired performance on the Corsi block-tapping task. The Corsi block-tapping task is a widely recognized measure of visuo-spatial working memory. Neuroimaging research has shown increased activity in the right hippocampus during Corsi block-tapping task performance compared to baseline, suggesting its role in encoding spatial locations^87^. Studies with stroke patients have also implicated bilateral hippocampal formation in maintaining spatial information over time^103^. Remarkably, performance on the Corsi block-tapping forward task was correlated with participants’ LDI scores. This observed correlation between Corsi block-tapping forward task performance and LDI scores suggests potential interactions between visuo-spatial working memory and hippocampus-dependent mnemonic processes. While the correlation aligns with the literature suggesting hippocampal involvement in both tasks, it does not conclusively establish that the observed deficits in mnemonic discrimination are solely hippocampal in origin. Executive functions, such as attentional control and working memory, mediated by prefrontal cortical regions, may also play a contributory role. However, our study found no other evidence of executive dysfunction. Tasks assessing inhibition (go/no-go, stop-signal task), flexibility (task-switching task), and updating (n-back task) revealed no significant differences between groups. This suggests that the correlation between Corsi block-tapping performance and LDI scores is unlikely to be driven by generalized executive deficits. Instead, the findings may reflect disruptions specific to hippocampal processes, including visuo-spatial working memory and mnemonic discrimination. Further research employing neuroimaging and more granular experimental designs is necessary to disentangle these overlapping cognitive and neural processes. Such studies could help determine whether COVID-19’s cognitive effects are predominantly mediated through hippocampal dysfunction, broader executive impairments, or an interaction between the two.

Our findings regarding the influence of SARS-CoV-2 infection on the questionnaire results provide important insights on the role of an infection on potential neuropsychiatric conditions related to depressiveness, general anxiety, and perceived stress, as well as well-being. Controlling for the demographic variables age, gender, and level of education allows for interpreting the observed differences between infection groups within demographic context. Multiple linear regression analyses showed that for the PHQ-9 (depressiveness), the effect of infection status remained significant even after controlling for age, gender, and level of education. This underscores the robustness of the association between infection history and depressive symptoms. Similarly, for the PSQ-20 (perceived stress) and WHO-5 (well-being), infection status continued to significantly predict scores after accounting for demographic variables. These results suggest that the impact of SARS-CoV-2 infection on mental health extends beyond the effects of demographic characteristics. However, for the GAD-7 (generalized anxiety), the effect of infection status was only marginally significant after controlling for these factors. The known relationship between lower education levels and higher anxiety^104^ and depressive symptom scores^84^ aligns with the significant educational differences observed between the infection groups in our sample. Although our results highlight the significant impact of infection status on mental health, they also point to the complex interplay between demographic factors and questionnaire scores. Nevertheless, the significant effects of infection status even after controlling for demographic factors underscore the unique contribution of a history of COVID-19 infection to mental health outcomes.

While our study offers valuable insights, it has several limitations. A key limitation of this study is the absence of direct structural or functional assessments of the hippocampus, which restricts our ability to draw definitive conclusions about hippocampal integrity. Our findings are based on behavioral measures, particularly the LDI, which is a widely used proxy for hippocampal function, especially for assessing mnemonic pattern separation processes associated with the dentate gyrus. While these measures provide indirect evidence of hippocampal involvement, they do not capture structural or functional changes directly. Consequently, alternative explanations for the observed deficits cannot be ruled out. For instance, disruptions in broader neural networks, or interactions with cortical regions might also contribute to the observed pattern of results. Although our analysis accounted for potential confounding factors like age, sex, and neuropsychiatric symptoms, the reliance on behavioral data alone limits the specificity of our conclusions about hippocampal dysfunction. To address these limitations, future studies should incorporate neuroimaging techniques, such as high-resolution fMRI or DTI, to directly assess hippocampal structure and function. These methods would provide a more detailed understanding of the neural mechanisms underlying the cognitive deficits observed in COVID-19 survivors and could help confirm whether the impairments are indeed localized to the hippocampus or involve broader neural circuits. Despite this limitation, our findings contribute to the growing body of evidence suggesting selective cognitive impairments post-COVID-19 and highlight the importance of further investigating hippocampus-dependent processes.

Another limitation of our study is that participants were not explicitly asked about the total number of SARS-CoV-2 infections they had experienced. While data collection occurred during a time when reinfections were relatively uncommon, this variable could provide important insights on the cumulative effects of repeated exposures on cognitive outcomes. Future research should include detailed data on the number and timing of infections to evaluate these effects in the context of evolving viral variants and changing immunity patterns. Moreover, for participants who had been infected multiple times, the “time since PCR” variable did not specify which infection was being referenced. This limitation could explain why impairment appears to increase with elapsed time since the positive PCR test – participants reporting their first infection might have experienced multiple infections in the interim, resulting in a high value for the “time since PCR” variable but also a high number of symptoms and a more severe mnemonic discrimination impairment. Consequently, their most recent infection might have occurred recently without our knowledge. Moreover, the time since PCR was measured only in months, which might overlook significant changes occurring in the days and weeks immediately following infection. Another factor with the potential to bias the assessment of recovery over time is the possibility that individuals reporting an infection several months ago might have participated for different motivational reasons than individuals participating in the acute phase. In particular, individuals with a long COVID history and possibly severe long-term symptoms might have been especially motivated to participate even months after infection, in contrast to individuals who have recovered shortly after infection. Consequently, not finding the LDI to recover over time might simply result from an overrepresentation of severely impaired individuals in the group whose participation was several months ago. Although we do not find the time elapsed between PCR test and study participation to be correlated with the number of sequelae, *r* = − .05,* t*(74) = 0.39, *p* = .695, we do not have a measure of perceived symptom severity. Additionally, the cross-sectional design restricts our ability to draw causal inferences, as it captures only a single point in time rather than the progression of changes. This design makes it difficult to determine whether observed impairments are directly caused by COVID-19 or influenced by other factors. Moreover, although participants in the group with an infection history reported having a PCR-confirmed positive result, we relied on self-reported data for COVID-19 test outcomes and the timing of their diagnosis. This reliance introduces potential inaccuracies, such as recall bias or discrepancies in reporting, which could affect our estimates of the prevalence and duration of COVID-related cognitive deficits. Participants may also not accurately remember or may underreport their symptoms and cognitive difficulties, potentially skewing the results. Future research should adopt longitudinal designs that follow participants over time. This approach would provide a clearer picture of the trajectory of cognitive and psychological recovery post-COVID-19 and help identify any long-term effects. Additionally, incorporating neuroimaging studies would allow researchers to investigate the underlying neural mechanisms. This could elucidate specific brain changes associated with the cognitive deficits observed, offering a more comprehensive understanding of the impact of COVID-19 on the brain. In addition to the limitations previously mentioned, our study also faced challenges inherent to online research. One significant limitation is the lack of control over the participants’ testing environment. Unlike in a laboratory setting, we cannot ensure that participants have a standardized and distraction-free environment, which could affect the accuracy and reliability of their responses. Moreover, the online format may introduce issues related to digital literacy and access. Participants with varying levels of familiarity with technology or those without reliable internet access may have faced difficulties in completing the study, potentially leading to sample bias and limiting the generalizability of the findings. Another concern is the difficulty in verifying participants’ identities and ensuring that they completed the tasks independently. This lack of oversight increases the risk of data integrity issues, such as participants providing inaccurate or inconsistent information. Lastly, online studies often rely on self-administered assessments, which can be less controlled compared to in-person testing. This may result in variations in how participants understand or engage with the tasks, potentially impacting the overall validity of the results.

One further limitation of this study is the decision not to apply formal corrections for multiple comparisons across the cognitive tasks. While such corrections are standard in confirmatory analyses, this study was designed with an exploratory focus to identify potential associations between prior COVID-19 infection and cognitive function. Apart from the hypothesis of an infection-related impairment in pattern separation (as measured by the LDI), the primary aim of our study was to generate hypotheses and highlight patterns warranting further investigation, rather than to provide definitive causal evidence. In exploratory contexts, where the primary concern is identifying potential associations rather than definitive significance, applying stringent corrections may lead to excessive conservatism and increase the risk of type II errors, potentially overlooking subtle but meaningful effects^105^. This would especially be the case in a large-scale study as reported here, in which the inclusion of multiple different, potentially informative tasks and analyses would be penalized by applying correction for an extensive number of comparisons. In consequence, the risk of over-correction would make the reported p-values almost meaningless. Rather than focusing on pure significance of effects, we recommend to set focus on effect sizes (see also^106^), which provide critical information on the magnitude of observed effects independent of sample size. Furthermore, similar studies in the field have also opted against strict multiple comparison corrections for these reasons, focusing instead on exploratory interpretation and hypothesis generation (e.g.,^68,107^, see also^108^). Moreover, the reported findings are interpreted within the framework of existing literature, which lends additional support to the plausibility of the observed effects^109^. We acknowledge that the lack of multiple comparison corrections may increase the risk of type I errors, and we strongly encourage readers to interpret the findings with this limitation in mind. However, the exploratory nature of this study necessitates a balance between type I and type II errors to ensure that potential effects are not prematurely dismissed. Future confirmatory studies are needed to replicate these findings under more stringent statistical conditions.

One of the primary strengths of our study is the detailed control for potential confounding variables such as age, level of education, sex, depression, anxiety, and stress. By accounting for these factors, we can more accurately isolate the specific cognitive effects attributable to COVID-19, enhancing the validity of our findings. Moreover, our study’s methodology allows for the detection of subtle cognitive deficits, which might be missed by more general cognitive screening tools often used in previous studies, like the Montreal Cognitive Assessment (MoCA) or the Mini-Mental State Examination (MMSE) or even standard word learning tests such as the California Verbal Learning Test (CVLT). These traditional tools are designed to identify significant cognitive impairments and might not be sensitive enough to pick up on the subtle cognitive changes that can occur even in an asymptomatic or mild course of COVID-19. Hence, by employing more refined cognitive assessments, we could identify and characterize mild deficits that could be overlooked in broader screenings. Another strength of our study is the broad participant spectrum. The inclusion of a wide range of COVID-19 survivors, including those without severe symptoms or prolonged neurological manifestations, provides a comprehensive view of the potential cognitive impact of COVID-19 across different severities of the illness. Our findings underscore that very specific cognitive reductions are not confined to hospitalized patients or patients who experienced severe prolonged neurological symptoms but may also be present in a less severe form among COVID-19 survivors and even in those who consider themselves fully recovered.

Given the ongoing nature of the COVID-19 pandemic, our study provides timely insights into the cognitive consequences of the virus, informing both clinical practice and future research directions. Understanding the full spectrum of COVID-19’s impact is crucial for developing effective interventions and support systems for affected individuals. Our study also highlights a critical public health issue by showing that even mild or asymptomatic COVID-19 cases can have lingering cognitive effects. This finding emphasizes the need for awareness and potential screening for cognitive issues in the broader population of COVID-19 survivors, which could lead to better management and support strategies. These findings also have several practical implications. First, they highlight the need for ongoing monitoring and support for individuals recovering from COVID-19, particularly those with persistent symptoms. Mental health services should be made readily available to address the elevated levels of anxiety, stress, and depression observed in those with a history of COVID-19. Furthermore, enhancing hippocampal neurogenesis could be particularly beneficial. Strategies to improve hippocampal neurogenesis include regular aerobic exercise, such as running or swimming, which has been shown to stimulate the growth of new neurons in the hippocampus^110–112^. Additionally, a diet rich in omega-3 fatty acids, antioxidants, and anti-inflammatory foods can support brain health, with foods such as fatty fish, nuts, berries, and leafy greens being particularly beneficial^113–115^. Techniques like mindfulness, meditation, and yoga can reduce stress^116^, which is known to negatively impact hippocampal neurogenesis^117^. Ensuring adequate and quality sleep is crucial for brain health and the promotion of neurogenesis^118^. Furthermore, certain medications have shown potential in stimulating neurogenesis, such as selective serotonin reuptake inhibitors (SSRIs) commonly used as antidepressants^119,120^, and other compounds like memantine, which is used in the treatment of Alzheimer’s disease^121,122^.

Our findings might not be specific to COVID-19 but could be common to other viral infections as well. Many viral infections can induce systemic inflammation and immune responses that adversely affect brain function and structure, particularly in regions like the hippocampus. For instance, other respiratory viruses, such as influenza and human coronaviruses, have demonstrated neuroinvasive capabilities, potentially causing encephalopathy and long-term neurological disorders^123,124^. These viruses can damage the central nervous system through direct viral replication or by triggering misdirected immune responses^124^. Additionally, the Zika virus has been shown to preferentially target neural stem cells, negatively affecting neurogenesis and synaptogenesis^125^. This evidence suggests that the cognitive impairments we observed may reflect a broader impact of viral infections on neurogenesis and cognitive function rather than being unique to COVID-19.

## Methods

### Participants

The link to the SosciSurvey^126^ (https://www.soscisurvey.de) online survey was distributed via social media, press releases, and the German Health Departments, which shared the link via their department-specific websites, social media, and flyers attached to the standard mail and/or e-mail to infected individuals. All participants gave written informed consent prior to participation. The study was approved by the local ethics committee of SRH University Heidelberg and Heidelberg University of Education [EV2021-09]. All methods were performed in accordance with the relevant guidelines and regulations. Participation was possible between July 15, 2021, and July 15, 2022.

Overall, *N* = 1405 adult participants (inclusion criterion was age > = 18 years; age range in the remaining sample: 18–90 years) were identified by their individual code, which they had generated at the very beginning of the survey, ensuring anonymity. While demographic data, information on infection status, health conditions, and participants’ responses to questionnaires were collected via SosciSurvey, another part of the study consisted of cognitive tasks that were run on the Pavlovia platform (https://pavlovia.org/) and were connected to the survey via external links. For the cognitive tasks, the sample characteristics differed from the overall sample described, as only subgroups of participants completed the various tasks, possibly due to motivational factors or because participants started the study using mobile devices without a physical keyboard. If participants took part in the survey multiple times in a row, only their first participation was considered for sample description and all analyses. According to their self-reported infection status, *n* = 495 participants had never had a positive COVID-19 PCR test result and *n* = 910 participants had received a positive test result in the past, irrespective of the time elapsed between the PCR test and their participation (*M* = 11.15 months, *SD* = 6.97, range = 0–29; 0 months indicates a positive PCR test within the last four weeks prior to participation). Infection groups (previously infected, previously not infected) did not differ regarding age, *t* < 1, and gender, χ² < 1 (see Table 5 for descriptives). In contrast, there was a significant association between infection status and level of education, χ² (1) = 13.56, *p* < .001. Specifically, individuals who had attained post-secondary education displayed a diminished prevalence of infections compared to their counterparts without completion of post-secondary education.

**Table 5:** Demographics of the overall sample (*N* = 1405), separately for participants previously infected and previously not infected.

Variable	Infection status		
	Previously not infected	Previously infected	Total
Age (in years)	*M* = 43.91 (*SD* = 14.93)	*M* = 44.06 (*SD* = 14.34)	*M* = 44.01 (*SD* = 14.54)
Gender			
Male	*n* = 133	*n* = 256	*n* = 389
Female	*n* = 361	*n* = 652	*n* = 1013
Diverse	*n* = 1	*n* = 2	*n* = 3
Education			
No post-secondary education	*n* = 278	*n* = 603	*n* = 881
Post-secondary education	*n* = 217	*n* = 307	*n* = 524
Employment			
Employed (incl. self-employed)	*n* = 378	*n* = 755	*n* = 1133
Job-seeking	*n* = 18	*n* = 23	*n* = 41
Retired	*n* = 58	*n* = 63	*n* = 121
Homemaker	*n* = 15	*n* = 21	*n* = 36
Other	*n* = 26	*n* = 48	*n* = 74
Body-Mass-Index (BMI)	*M* = 26.14 (*SD* = 5.54)	*M* = 26.53 (*SD* = 5.45)	*M* = 26.39 (*SD* = 5.49)

Post-secondary education indicates if participants have completed some post-secondary education (i.e., any degree higher than high-school diploma) or not (see^127^). BMI is calculated as weight in kg/((height in m)²), underweight < 18.5, normal range = 18.5-24.99, overweight 25-29.99, obesity > 30 (World Health Organization [WHO]^128^).

When participants self-reported a positive result on a PCR test, they were subsequently asked about their vaccination status preceding the positive test result (vaccinated, not vaccinated). This question was not mandatory and added to the survey only in November 2021. Furthermore, participants affirming a positive test were further prompted to delineate their acute COVID-19 symptoms at the time of infection, enduring COVID-19 symptoms, and long COVID symptoms.

### Measures

Participants reached the study via a hyperlink to the SosciSurvey platform. After giving written informed consent and completion of the questions regarding demographic data, infection status, vaccination status, and both acute and post-acute COVID-19 symptoms, we assessed depressiveness, stress, anxiety, and well-being (always in this order) using the Patient Health Questionnaire (PHQ-9), the Perceived Stress Questionnaire (PSQ-20), the Generalized Anxiety Disorder Scale (GAD-7), and the WHO well-being (WHO-5) questionnaires, respectively. Next, participants were asked for information on their health condition to exclude participants with known physical or mental disorders that might affect performance in the cognitive tasks (see Analyses section). For the same reason, regular medication intake was assessed through an open-format question in which participants were asked to provide the names of any medications they were taking regularly. This approach allowed for the detailed and unrestricted reporting of medication use, ensuring comprehensive data collection for this variable.

**Fig. 1:**
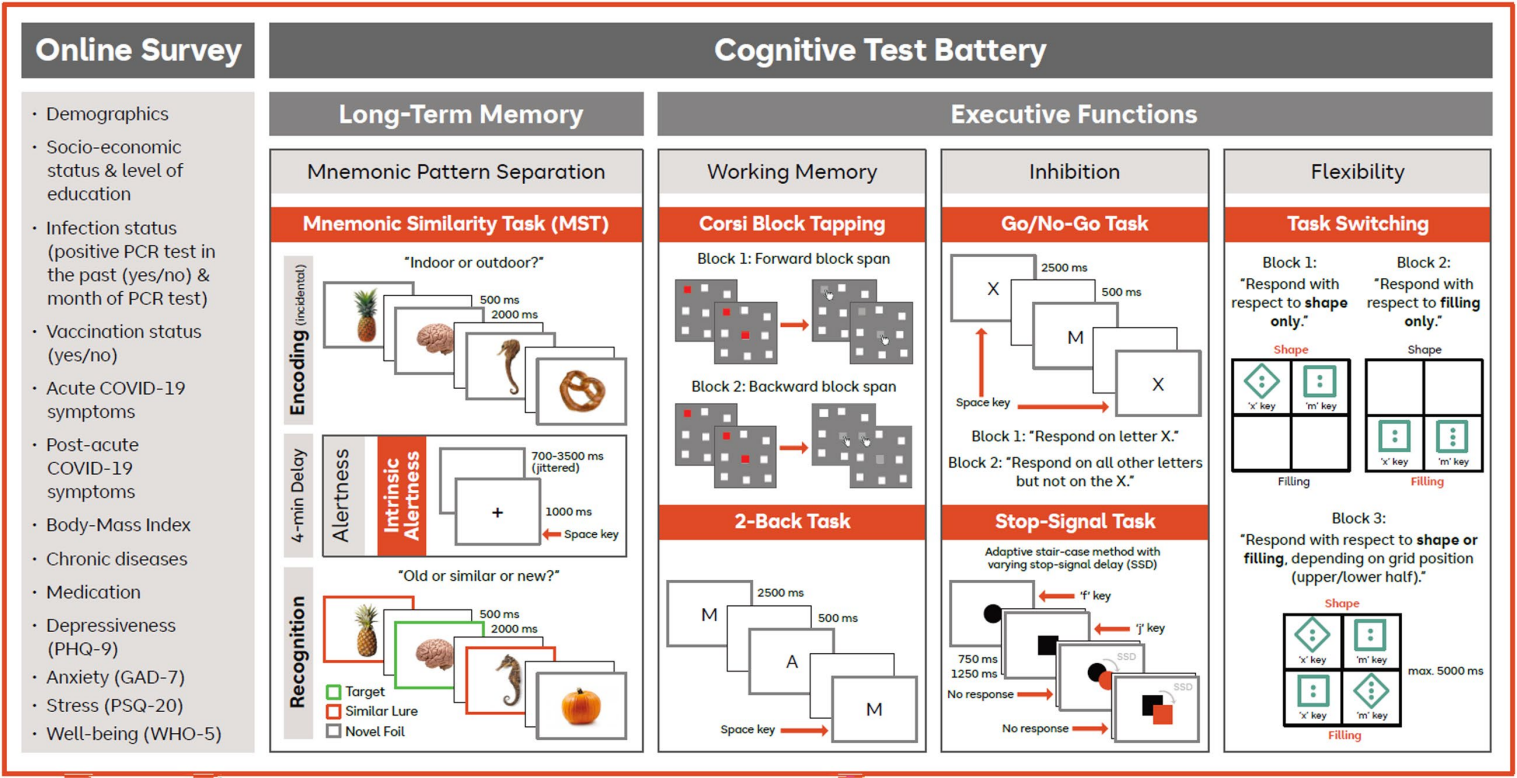
Design and procedure of the online survey and the cognitive test battery. Tasks of the cognitive test battery were applied in random order, with the restrictions that (1) the alertness task was always inserted between the study and test phase of the mnemonic similarity task (MST) and that (2) the Corsi block-tapping forward task was always conducted before the backward version. MST pictures were drawn from the publicly available Stark lab github repository, with random assignment to Sets 1–6 (https://github.com/celstark/MST).

The survey part of the study was followed by the cognitive test battery (see Fig. 1). All cognitive tasks were programmed in PsychoPy^129^ (https://www.psychopy.org/) and run on the Pavlovia platform (https://pavlovia.org/). To match the survey datasets with the cognitive task data, participants generated an individual code at the beginning of the study, which they were prompted to fill in at the beginning of every cognitive task. The overall eight cognitive tasks automatically started in full-screen mode by clicking on the respective Pavlovia link. Cognitive tasks were administered in random order, with the constraints that the Corsi block-tapping backward task was always preceded by the Corsi block-tapping forward task and that the alertness task was always used as a distractor task between the study and the test phase of the MST.

#### Questionnaires

##### Depressiveness

To assess depressive symptoms within the previous two weeks, we administered the Patient Health Questionnaire (PHQ-9^79^). Participants were asked to indicate the frequency of their experience with each of nine depressive symptoms (four-point scale: “not at all”, “several days”, “more than half the days”, “nearly every day”, scored as 0 to 3, respectively). This scoring system yielded a potential total score ranging from 0 to 27. PHQ-9 scores allow for a stratification of depression severity, with classifications as follows: minimal: 0–4, mild: 5–9, moderate: 10–14, moderately severe: 15–19, severe: ≥ 20).

##### Perceived stress

To evaluate perceived stress within the concurrent two-week timeframe, aligning with the temporal scope of the other administered questionnaires, an adapted version of the Perceived Stress Questionnaire (PSQ-20^80^) was employed. This modification involved a temporal adjustment, focusing on the preceding two weeks instead of the original four-week duration. The PSQ-20 comprises four distinct subscales, namely demands, tension, joy (with reverse coding in total score), and worries, containing five items each. Participants were asked to rate how often an item applies to their experience within the last two weeks using a four-point scale (“almost never”, “sometimes”, “often”, “usually”, scored as 0 to 3, respectively). Mean scores of the subscales were divided by 3 and multiplied by 100, resulting in a mean subscale score that ranges from 0 to 100. The total score represents the mean of the transformed subscale scores.

##### Anxiety

The Generalized Anxiety Disorder Scale (GAD-7^81^) was employed to assess the frequency of seven symptoms associated with general anxiety. Participants rated their experiences on a four-point scale ranging from: “not at all”, “several days”, “more than half the days”, to “nearly every day”, scored as 0 to 3, respectively, resulting in a possible total score range between 0 and 21). GAD-7 scores allow for classification based on anxiety levels, with scores exceeding 10 falling within the clinical range (mild: 5–9, moderate: 10–14, severe: ≥ 15).

##### Well-being

To assess the overall well-being of our sample, we used the WHO well-being index (WHO-5^82^; see also ^83^). Participants were asked to report the frequency of experiencing five different states reflecting high levels of well-being within the last two weeks using a six-point scale ranging from “never” to “all the time” (scored as 0 to 5). Raw scores were then transformed into a total score (i.e., multiplied by 4) covering a range from 0 to 100. Scores ≤ 50 are considered indicators of a poor emotional state, warranting further examination^130^.

#### Cognitive tasks

##### Long-term memory

The Mnemonic Similarity Task (MST^67^), a modified object recognition memory task, is designed to be highly sensitive to hippocampal function, particularly by imposing robust demands on pattern separation. Widely adopted in behavioral research, the MST has consistently demonstrated sensitivity to age-related memory decline, hippocampal connectivity, and overall hippocampal function, with a specific emphasis on the DG (see^ 67^, for an overview). This task has proven extremely useful in identifying hippocampal dysfunction across various conditions, including healthy aging, dementia, schizophrenia, depression, and other clinical disorders^87,131^. For instance, in amnesic patients with lesions limited specifically to the hippocampus, impaired lure discrimination performance has been observed, while item recognition performance remains intact^132^. Further supporting the role of the DG in mnemonic pattern separation, a rare case with damage isolated to the DG displayed selective impairments in discriminating similar lures, while recognition performance remained normal^133^. Similar findings have been reported in patients with CA1-specific lesions, a crucial output structure of the DG^134^. Extending beyond clinical populations, studies involving healthy individuals have revealed that pattern-separation performance as measured by the MST reflects hippocampal activity, distinguishing between accurately discriminated lures and instances where the lure is mistakenly identified as old^135,136^. This discrimination ability can be localized to some extent to the DG/CA3 regions within the hippocampus^137^.

The MST comprises an incidental picture encoding phase followed by an unexpected recognition memory assessment (see Fig. 1). All pictures used in the MST were drawn from the openly available stimulus pool provided on the Stark lab github repository (https://github.com/celstark/MST). Participants were randomly assigned to one of six stimulus sets (Set 1–6 of the stimulus pool), each containing 384 color photographs of objects organized into 192 pairs that consist of two similar items each. Before the study phase, the stimulus pairs were randomly shuffled, individually for each participant. During the study phase, participants were presented with 128 items drawn from these unique pairs. At the subsequent memory test, 64 of these objects were reintroduced as old items (targets). The remaining previously presented objects had their respective similar item presented at test (lures). In addition, the 64 objects of the stimulus pool that had not been encoded in the study phase were presented as new items (foils), resulting in 192 recognition test trials overall.

At the beginning of each of the 128 study-phase trials, a fixation cross was presented in the center of the screen for 500 ms, which was then replaced by an object picture for 2000 ms. For as long as the picture was presented, participants could indicate that they think the object typically appears indoor or outdoor by pressing the ‘f’ or ‘j’ key on their computer keyboard (counterbalanced between subjects), with key assignment reminders displayed at the bottom of the screen throughout the encoding phase. Following the study phase, a simple 5-minute stimulus-response alertness task was administered as a filler task (see below). During the subsequent recognition test phase, participants encountered the 192 pictures as described earlier (targets, lures, foils; see Fig. 1) in random order. Each trial began with the presentation of a fixation cross in the center of the screen for 500ms, followed by the presentation of the object for 2000 ms. The participants’ task was to indicate whether the objects were old, similar, or new by pressing the ‘c’, ‘b’, or ‘m’ key on the computer keyboard, respectively. Responding was possible for as long as the picture was presented.

##### Alertness

The alertness task closely followed the procedure of the TAP (Testbatterie zur Aufmerksamkeitsüberprüfung^138^) Alertness test part assessing intrinsic alertness. In each trial, participants were presented with a black fixation cross against a white background in the center of the screen for 1000 ms (see Fig. 1). Their task was to press the space key as fast as possible as soon as the fixation cross appeared. Inter-stimulus intervals were jittered ranging from 700 to 3500 ms in steps of 100 ms. These intervals were randomly selected from a pool comprising all 29 jitter durations. The task was organized into four blocks of 20 trials each. The blocks were separated by 8-second breaks, resulting in an overall duration of approximately 5 min.

##### Executive functions

**Working Memory**

*Corsi block tapping*

We assessed visuo-spatial working memory utilizing an online adaptation of the Corsi block-tapping task (inspired by the digital version outlined in Arce & McMullen^139^), primarily derived from the online Corsi block-tapping demo accessible via https://gitlab.pavlovia.org/demos/corsi). Each participant engaged in both a forward and a backward version of the Corsi block-tapping task, consistently following this order. In both versions, participants were presented with nine randomly positioned, non-overlapping white squares on grey background. Their task was to memorize a random sequence of squares, visually indicated by sequentially highlighting the blocks. Specifically, a square changed its color to red for one second before reverting to white, and the subsequent square in the sequence turned red (see Fig. 1). After completion of a sequence, participants were instructed to replicate the sequence by clicking on the blocks either in the original order (forward version) or in reverse order, starting with the last highlighted block (backward version), without any time constraints. A tapped square transitioned to light grey and remained so until the participant’s number of clicks matched the length of the sequence. After completing the required number of taps of the respective sequence (regardless of success), a 500 ms inter-trial interval was inserted, in which a fixation cross was presented, before the next trial started. In both the forward and backward version, the length of the block sequence progressively increased during the task. Starting with a sequence length of three blocks, participants completed five trials at each length before the sequence extended by one block. This process continued until reaching a maximum sequence length of nine blocks. If a participant failed to reproduce a sequence correctly in at least three out of the five trials, subsequent sequence lengths were presented only once. This approach ensured comparable task familiarization and interference levels for all participants, irrespective of their success, particularly as the performance in the backward version immediately following should not be influenced by varying degrees of task exposure in the forward version.

*N-back task*

We assessed visual working memory using a 2-back version of the well-established n-back task^140^. In this task, participants were sequentially presented with single letters and were instructed to press the space key whenever the current letter matched the one shown two trials before (i.e., n-back trials). A set of twenty-four letters was randomly selected from a pool of 16 unique letters (A, B, C, D, F, G, H, J, K, L, M, N, P, S, T, and U). These letters were displayed in black Arial font against a white background (see Fig. 1). Each trial started with a fixation cross that was presented at the center of the screen for 2000 ms. Subsequently, the fixation cross was replaced by the randomly chosen letter, which remained on the screen until a response was made or for a maximum of 500 ms. Participants could respond throughout letter presentation plus an additional 500 ms after the letter was no longer visible. The entire task consisted of 96 trials, organized into four blocks separated by a 10-second break. Each block comprised 24 trials, including six n-back trials.

**Flexibility**

To assess the costs associated with switching between two different tasks in comparison to conditions in which only one task was performed^141^, the task-switching test encompassed three different phases (primarily derived from the online task-switching experiment accessible via https://gitlab.pavlovia.org/c8b/task-switching). The first two phases were task-homogeneous, meaning participants engaged in a single task, while the third phase was task-heterogeneous, necessitating participants to flexibly switch between two tasks. In both tasks, a grid of black lines dividing a square into four white quarters was centrally presented on the screen (see Fig. 1). Each trial involved the appearance of either a square or diamond shape outlined by a green frame, filled with green dots. A total of four combinations of shapes and fillings could occur in one quarter at a time (i.e., square comprising two dots, square comprising three dots, diamond comprising two dots, diamond comprising three dots). In the task-homogeneous shape task, diamonds and squares filled with two or three dots exclusively appeared in the upper half of the grid. Participants were instructed to respond with respect to the shape (i.e., press the “x” key for diamonds, press the “m” key for squares) and ignore the filling. Conversely, in the task-homogeneous filling task, stimuli consistently appeared in the lower half of the grid. Participants were instructed to respond with respect to the filling (i.e., press the “x” key for two dots, press the “m” key for three dots) and ignore the shape. During the task-heterogeneous phase, both tasks were integrated: Participants were instructed to respond with respect to shape when a stimulus appeared in the top half of the grid and to decide based on the filling when a stimulus appeared in the bottom half. Half of the task-heterogenous trials were repeat trials (e.g., a “filling” trial preceded by another “filling” trial) and the other half were switch trials (e.g., a “filling” trial preceded by a “shape” trial). The experimental phase comprised 28 task-homogeneous shape-only trials, 28 task-homogeneous filling-only trials, and 52 task-heterogeneous trials (26 switch trials, 26 repeat trials). Four buffer trials at the beginning of each phase were not included in the analyses, resulting in 24, 24, and 48 valid trials, respectively. In a preceding practice phase, participants completed 8 trials in the shape-only condition, 8 trials in the filling-only condition, and 16 trials in the task-heterogeneous condition (8 repeat trials, 8 switch trials).

**Inhibition**

*Go/no-go task*

The go/no-go task serves as a measure of inhibition, assessing the ability to withhold responses to a specific stimulus (no-go stimulus), while responding to other, more frequent stimuli (go stimuli). All stimuli in this task were black letters presented in Arial font against a white background in the center of the screen (see Fig. 1). In the first of two phases, the letters E, F, H, K, M, N, T, V, W, and Y were used as no-go stimuli, each presented twice, resulting in 20 no-go trials. The go stimulus X was presented 80 times. To intensify inhibition demands and elevate between-task interference (as in^142^), the second phase reversed the response instructions, with X serving as the no-go stimulus and the letters A, E, F, H, I, K, M, N, R, T, V, W, Y, and Z as go stimuli. Ten of these letters were presented six times and five letters were presented four times. This resulted in a total number of 80 go trials, aligning with the go/no-go trial ratio from Phase 1. In both phases, participants were instructed to respond to the *go* stimuli by pressing the space key and to refrain from responding in *no-go* trials. Letters were sequentially presented for 300 ms, following the procedure used by Redick et al.^142^. Responses could be made throughout stimulus presentation plus 700 ms after the letter had disappeared, resulting in a total response window of 1000 ms. Participants did not receive feedback. Participants could familiarize with the task in a practice phase consisting of 12 go trials and three no-go trials. In each practice trial, a letter was presented for 400 ms and responses could be made throughout the presentation plus further 1000 ms. Feedback on response accuracy was provided for 400 ms after each practice trial.

*Stop-signal task*

In the stop-signal task, participants were required to stop an already initiated response. Each trial started with the presentation of a black fixation cross against a white background in the center of the screen. After 250 ms, the fixation cross was replaced by either a black square or circle (see Fig. 1). Participants were asked to indicate which shape they saw as fast as possible by keypress (“f” for circles, “j” for squares). Each shape was presented in 50% of the overall 192 trials. Trials were organized in three blocks, each consisting of 64 trials (48 *go* trials, 16 *stop* trials; see^143^), with a 10-second break between blocks. In *go* trials, the stimulus remained on the screen for 1250 ms and the default inter-stimulus interval was fixed at 2000 ms, regardless of participants’ response latencies. In *stop* trials, the black shape turned red, signaling participants to inhibit their response. During these stop trials, participants were required to inhibit their initial *go* reaction and refrain from keypress. In stop-signal tasks, the delay between the onset of the go signal and the onset of the stop signal (stop-signal delay, SSD) determines the difficulty level of inhibiting an initiated response since inhibition becomes easier with shorter SSDs. The stop-signal task was programmed in an adaptive manner with a varying SSD, employing the adaptive staircase tracking algorithm of the STOP-IT software^144^. In particular, the SSD began at 250 ms in the first stop trial and continuously adjusted, based on inhibition success in the previous stop trial. Specifically, the SSD increased by 50 ms after successful inhibition and decreased by 50 ms after inhibition failure^144^. Consequently, the probability of a response given a stop signal was expected to be at 50%. In that way, the strategy to intentionally prolong one’s response latencies to facilitate inhibition would not be successful.

### Analyses

All analyses were conducted using R^145^. Regardless of the specific tasks, participants under the age of 18 were excluded from all analyses. In cases where participants engaged in the survey or completed cognitive tasks multiple times consecutively, we ensured that only the initial datasets were included in the analyses. In particular, only their initial dataset from the respective cognitive task was considered for analysis and this was only the case if this dataset could be uniquely matched with their first participation in the survey. For the analyses of the cognitive tasks, individuals lacking normal or corrected-to-normal vision were excluded. Additionally, participants with a history of critical health conditions, such as a stroke, cardiac arrest, cancer, or a brain surgery, as well as those who underwent major surgery within the last month or experienced a loss of consciousness for more than five minutes in the past, were excluded from the analyses. Further exclusions encompassed participants reporting diseases that could impact cognitive functioning, including psychiatric or neurologic disorders (e.g., narcolepsy, schizophrenia, epilepsy, diagnosed depression, migraine), untreated hypertension, and those under medication that could influence cognitive functioning (e.g., neuroleptics, antidepressants).

For all cognitive task analyses, a linear regression model was employed. Infection status (previously infected, previously not infected) served as fixed effect, while controlling for the covariates age (mean-centered), gender (male, female), level of education (binary as “high” and “low”, i.e., completed vs. no completed post-secondary education; see^146^, depressiveness (PHQ-9), anxiety (GAD-7), and stress (PSQ-20). Gender was coded as binary variable in the analyses of all cognitive tasks, as the three individuals with diverse gender did not participate in any of the tasks. All *p* values are reported two-tailed. Effect size *d* for independent t-tests comparing groups with and without a history of infection was calculated as the difference between group means of the dependent variable, divided by the pooled standard deviation. As the absence of evidence does not constitute evidence for the absence of an effect (see, e.g^146–148^), we additionally calculated Bayes Factors (BF) to better quantify the strength of evidence for or against the null hypothesis. For the regression analyses, we report BF_10_ values, representing the ratio of the likelihood of the data under the full model (including the effect of interest) to that under a reduced model without the effect. These were computed using the BayesFactor package for R^149^. For t-tests, BF_10_ were calculated using a Cauchy prior on effect size scaled at r = $\:\sqrt{2}/2$, indicating the degree of support for the alternative hypothesis (H_1_) over the null hypothesis (H_0_).

To interpret the resulting Bayes factors, we followed established classification schemes^150,151^. Specifically, BF_10_ values greater than 10 were considered to reflect strong to very strong evidence for H_1_, values between 3 and 10 indicated moderate evidence for H_1_, and values between 1 and 3 were taken as anecdotal evidence for H_1_. A BF_10_ around 1 suggested no diagnostic value in favor of either hypothesis. Conversely, values between 1/3 and 1 were interpreted as anecdotal evidence for H_0_, values between 1/10 and 1/3 as moderate evidence for H_0_, and values smaller than 1/10 were considered strong to very strong evidence for H_0_.

#### Long-term memory

##### Mnemonic similarity task

After excluding participants who met the general exclusion criteria outlined above, we further excluded participants who did not press each key in the recognition test at least once, as this suggests a potential misunderstanding of task instructions. The final datasets all contained a minimum of 10 trials per item category (target, lure, foil), resulting in a sample size of *N* = 151 (*n* = 86 previously infected, *n* = 65 previously not infected; age: *M* = 43.36, *SD* = 13.83, range = 18–69 years; 101 female, 50 male; *n* = 68 with completed post-secondary education, *n* = 83 without completed post-secondary education). On average, participants in this sample did not respond in 10.53 trials within the given response-time window. Infection groups (previously infected, previously not infected) did not differ in terms of age, *t* < 1, gender, χ² (1) = 1.97, *p* = .160, or level of education, χ² (1) = 1.95, *p* = .163. Within the subgroup of previously infected participants, the average time between the positive PCR test and study participation was 9.27 months (*SD* = 6.36, range: 0–25 months), with 13 participants having participated within three months after testing positive. Among the remaining 73 participants with positive test results of more than three months ago, 45 reported experiencing at least one post-acute/long-COVID symptom.

To assess mnemonic pattern separation, we computed each participant’s lure discrimination ability, quantified by the Lure Discrimination Index (LDI). The LDI indicates the ability to correctly identify lure items as lures, or, in other words, to recognize that the lures are similar (but not identical) to a studied item, corrected for a response bias towards accepting foils as lures. Thus, the LDI is calculated as the ratio of “similar” responses to lures minus the ratio of “similar” responses to foils, that is, *P*(“similar” | lure) – *P*(“similar” | foil). As an indicator of item recognition memory, we calculated the pr score as recognition index (REC). The REC index represents the ability to recognize target items as old while correcting for a bias towards endorsing foils as targets, that is, *P*(“old” | target) – *P*(“old” | foil). For all analyses of the MST data, participants with LDI or REC values beyond two standard deviations from the mean were excluded (see also^87^), separately for the previously infected and previously not infected group (*n* = 10 previously infected; *n* = 3 previously not infected). This resulted in a final sample size of *N* = 138 participants (*n* = 76 previously infected, *n* = 62 previously not infected). On a trial level, responses faster than 100 ms were excluded to prevent the results from being biased by accidental key presses (5.31% of all trials; see^152^). Additionally, a pattern completion (PC) bias reflecting the tendency to respond “old” to lure items was calculated as the ratio of “old” responses to lures minus the ratio of “similar” responses to lures, that is, *P*(“old” | lure) – *P*(“similar” | lure).

#### Alertness

Following the application of the exclusion criteria specified in the general Analyses section, we further excluded participants who failed to respond in at least 50% of the trials (*n* = 4, with a mean percentage of 7.50% trials with a response), resulting in a sample size of *N* = 155 (*n* = 92 previously infected, *n* = 63 previously not infected; age: *M* = 43.60, *SD* = 14.37, range = 18–76 years; 105 female, 50 male; *n* = 67 with completed post-secondary education, *n* = 88 without completed post-secondary education). Infection groups did not differ in terms of age, *t* < 1, gender, χ² < 1, and level of education, χ² = 1.16, *p* = .281. The primary outcome measure of this task were mean response latencies, with accuracy (i.e., hit rate, reflecting the proportion of correct responses on the signal relative to the overall trial number) also reported. In all analyses of the alertness data, participants whose dependent variables of interest (i.e., response latencies, accuracy) deviated by more than two standard deviations from the mean were excluded, separately for the previously infected and previously not infected group. For the response latency analyses, the removal of eight outlier participants (*n* = 4 previously infected; *n* = 4 previously not infected) resulted in a final sample size of *N* = 147 participants (*n* = 88 previously infected, *n* = 57 previously not infected). In accuracy analyses, six outlier participants were excluded (*n* = 4 previously infected; *n* = 2 previously not infected), resulting in a final sample size of *N* = 149 participants (*n* = 88 previously infected, *n* = 61 previously not infected).

#### Executive functions

##### Working memory

*Corsi block tapping*

In addition to the general exclusion criteria (see above), we excluded participants who were unable to accurately repeat even the initial sequence level (i.e., sequences of three blocks in at least three out of five trials). This precaution was taken to address any potential comprehension issues with the task. Overall, *N* = 250 participants were included in the analyses of the forward version (*n* = 192 previously infected, *n* = 58 previously not infected; age: *M* = 43.80, *SD* = 13.65, range = 18–72 years; 166 female, 84 male; *n* = 101 with completed post-secondary education, *n* = 149 without completed post-secondary education) and *N* = 224 participants in the analyses of the backward version (*n* = 176 previously infected, *n* = 48 previously not infected; age: *M* = 43.71, *SD* = 13.64, range = 18–72 years; 150 female, 74 male; *n* = 95 with completed post-secondary education, *n* = 129 without completed post-secondary education). Infection groups did not differ regarding age, *t* < 1, or gender, χ² < 1, in either task version. However, there was an uneven distribution of education levels across infection groups, χ² (1) = 4.66, *p* = .031, with a greater proportion of individuals with lower educational level in the group previously infected (*n* = 70 with completed post-secondary education, *n* = 122 without completed post-secondary education) compared with the group previously not infected (*n* = 31 with completed post-secondary education, *n* = 27 without completed post-secondary education). In the backward version, a similar pattern was observed, reaching marginal significance, χ² (1) = 2.87, *p* = .090, again with more individuals with a lower educational level in the group previously infected (*n* = 69 with completed post-secondary education, *n* = 107 without completed post-secondary education) than in the group previously not infected (*n* = 26 with completed post-secondary education, *n* = 22 without completed post-secondary education). Within the previously infected sample, the time between the positive PCR test and participation in the forward Corsi task was 11.34 months on average (*SD* = 7.10, range: 0–29 months) with 25 participants participating within three months of testing positive. Among the remaining 167 participants who had previously tested positive and whose infection was diagnosed more than three months ago, 112 reported experiencing at least one post-acute/long-COVID symptom. For the regression analyses, an additional participant (59 years old, previously infected) was excluded from the analyses of both task versions as he had not completed the questionnaires on depressiveness, anxiety, and stress.

The primary outcome measure for both Corsi versions was the maximum sequence length (i.e., block span) that could be reproduced correctly in at least three out of five trials, calculated separately for the forward and backward version.

*N-back task*

After applying the exclusion criteria outlined in the general Analyses section, we further excluded one participant who did not press the space key at least once, suggesting a potential misunderstanding of the task. In total, *N* = 328 participants were included in the analyses (*n* = 208 previously infected, *n* = 120 previously not infected; age: *M* = 41.94, *SD* = 13.92, range = 18–80 years; 223 female, 105 male; *n* = 146 with completed post-secondary education, *n* = 182 without completed post-secondary education). No significant differences emerged between infection groups in terms of age, *t* < 1, or gender, χ² < 1. However, there was an uneven distribution of education levels across infection groups, χ² (1) = 4.39, *p* = .036, with a higher proportion of individuals exhibiting lower educational levels in the group previously infected (*n* = 83 with completed post-secondary education, *n* = 125 without completed post-secondary education) compared with the group previously not infected (*n* = 63 with completed post-secondary education, *n* = 57 without completed post-secondary education). The primary outcome measure for assessing working memory performance was accuracy, quantified by the sensitivity measure *d*’ (i.e., *z*(Hit rate) – *z*(False Alarm rate)). To avoid *d*’ being undetermined in subjects with hit rates or false alarm rates equal to 0 or 1, we replaced scores equal to 0 by 0.5/*n* and scores equal to 1 by (*n*-0.5)/*n*, with *n* representing the total number of trials of the respective target category (i.e., target trials or foil trials; see also^153–155^). Participants identified as outliers within their group based on accuracy (i.e., above or below two standard deviations of the mean) were excluded from all analyses (*n* = 10 previously infected, *n* = 7 previously not infected). In addition to a linear regression model testing the impact of infection status on accuracy, the same model was also applied to mean response latencies for correct target responses (i.e., hits). For the regression analyses, an additional participant (59 years old, previously infected) was excluded from the analyses as he had not completed the questionnaires on depressiveness, anxiety, and stress. On the trial level, we excluded trials with response latencies less than 100 ms (0.04% of all trials).

##### Flexibility

In addition to the exclusion criteria outlined in the general Analyses section, seven participants were excluded due to incomplete participation in the initial two (task-homogeneous) experimental phases, as well as failing to complete a minimum of 10 switch trials and 10 repeat trials within the subsequent third (task-heterogeneous) phase. Overall, *N* = 325 participants could be included in the analyses (*n* = 216 previously infected, *n* = 109 previously not infected; age: *M* = 42.54, *SD* = 13.72, range = 18–69 years; 222 female, 103 male; *n* = 138 with completed post-secondary education, *n* = 187 without completed post-secondary education). Demographic comparisons indicated no significant differences in age between infection groups, *t* < 1, or gender distribution, χ² < 1. However, an uneven distribution was observed for educational level across infection groups, χ² (1) = 7.11, *p* = .008, with a notably higher proportion of individuals with lower educational levels in the group previously infected (*n* = 80 with completed post-secondary education, *n* = 136 without completed post-secondary education) compared with the group previously not infected (*n* = 58 with completed post-secondary education, *n* = 51 without completed post-secondary education). On average, participants in this sample did not respond in 4.51 trials within the given response-time window. Cognitive flexibility was assessed through two types of costs, namely (1) slowed responses and (2) increased error rates. Higher costs in both aspects indicate reduced flexibility. *Specific switch costs* were computed by subtracting repeat-trial response latencies (or error rates) in the task-heterogeneous block from switch-trial response latencies (or error rates) in the same block. *Mixing costs* were determined by subtracting response latencies (or error rates) in the task-homogeneous blocks from non-switch trial response latencies (or error rates) in the task-heterogeneous block. Error rates represented the proportion of incorrect responses within a trial category. Response latencies were log-transformed for the regression analyses to meet the requirement of normally distributed residuals. On the subject level, we further excluded participants who were identified as outliers within their group regarding the respective cost effect (i.e., above or below two standard deviations of the mean). For the costs in response latencies, we excluded 16 outliers for the specific switch-cost analyses (*n* = 11 previously infected, *n* = 5 previously not infected) and 15 outliers in the mixing-cost analyses (*n* = 10 previously infected, *n* = 5 previously not infected). For the analyses of error costs, we excluded 15 outliers (*n* = 10 previously infected, *n* = 5 previously not infected) for the specific switch-cost analyses and 19 outliers (*n* = 13 previously infected, *n* = 6 previously not infected) for the mixing-cost analyses. On the trial level, trials with response latencies of < 100 ms were excluded from all analyses (1.44% of all trials). For regression analyses, two participants (20 years old, previously not infected, and 59 years old, previously infected) were excluded due to incomplete questionnaires on depressiveness, anxiety, and stress.

##### Inhibition

*Go/no-* *go task*

In addition to the general exclusion criteria (see above), six participants were omitted from the analyses (*n* = 4 previously infected, *n* = 2 previously not infected) due to their failure to complete a minimum of 10 go and 10 no-go trials within each phase. Consequently, the final sample comprised *N* = 427 participants (*n* = 219 previously infected, *n* = 111 previously not infected; age: *M* = 43.45, *SD* = 14.14, range = 18–82 years; 237 female, 93 male; *n* = 136 with completed post-secondary education, *n* = 194 without completed post-secondary education). Comparisons between infection groups revealed no significant differences in age, *t* < 1, or gender distribution, χ² < 1. However, the distribution of educational levels was uneven across infection groups, χ² (1) = 4.29, *p* = .038, indicating a disproportionate number of individuals with lower educational levels in the group previously infected (*n* = 81 with completed post-secondary education, *n* = 138 without completed post-secondary education) compared with the group previously not infected (*n* = 55 with completed post-secondary education, *n* = 56 without completed post-secondary education). To assess the ability to inhibit responses in no-go trials we focused on the false-alarm rate (i.e., incorrect responses on no-go trials). Sensitivity, represented by *d*’ (i.e., *z*(Hit rate) – *z*(False Alarm rate)), was calculated to account for response bias. The rationale behind this measure posited that, in the event of inhibition impairment caused by a prior SARS-CoV-2 infection, previously infected participants would exhibit a decrease in *d*’ particularly driven by an increase in false alarm rate. For all participants irrespective of infection status, a decrease in *d*’ should be observed between the first and the second test phase. To avoid *d*’ being undetermined in case of hit rates or false alarm rates equal to 0 or 1, scores equal to 0 were replaced by 0.5/*n* and scores equal to 1 were replaced by (*n*-0.5)/*n*, with *n* representing the total number of trials in the respective trial category (i.e., go trials or no-go trials; see also^153–155^). Outliers with respect to *d*’ (i.e., above or below two standard deviations of the mean), identified separately for their infection status groups (*n* = 19 previously infected, *n* = 9 previously not infected), were removed.

*Stop-signal task*

Performance in the stop-signal task was modeled as a race between two different processes: a *go* process initiated by the *go* signal and a *stop* process activated by a *stop* signal. Following the presentation of both signals, these processes operate concurrently, and the outcome depends on which process finishes first – whether responses are successfully inhibited or not. Successful inhibition is characterized by the completion of the stop process before the go process finishes. Conversely, if the go process finishes before the stop process, participants are unable to inhibit their response. Measuring response inhibition constitutes a unique challenge due to the absence of participant reactions in (correct) response-inhibition trials. However, the stop-signal reaction time (SSRT) can be mathematically estimated based on an independent race model^156^. According to this model^157^, SSRT is calculated as the difference between stop-signal delay (SSD; i.e., the time between the onset of the go signal and the onset of the stop signal) and the time when the internal response to the stop signal occurs. For the adaptive staircase procedure we used in this task, the internal response to the stop signal equals the mean go reaction time as participants should necessarily stop half of their responses in the stop trials^158^. However, the proportion of commission errors (i.e., false alarms in stop trials) in participants who respond extremely rarely or extremely often can deviate from 50%. Therefore, participants with < 25% or > 75% commission errors or > 10% omission errors (i.e., no response in go trials) were excluded (*n* = 49 previously infected, *n* = 29 previously not infected), following recommendations by Verbruggen et al.^143^  (see also^144,159^). Moreover, remaining participants whose response latencies on unsuccessful stop trials were numerically longer than those on go trials were also excluded, as SSRT cannot be reliably estimated in such cases[143] (*n* = 6 previously infected, *n* = 1 previously not infected;). After removing outliers regarding SSRT (two standard deviations above or below the mean of the respective infection group; *n* = 8 previously infected, *n* = 5 previously not infected), the final sample consisted of *N* = 245 participants (*n* = 167 previously infected, *n* = 78 previously not infected), age: *M* = 43.09, *SD* = 13.93, range = 18–80 years; 167 female, 78 male; *n* = 117 with completed post-secondary education, *n* = 128 without completed post-secondary education). No significant differences between infection groups were observed regarding age, *t* < 1, or gender distribution, χ² < 1. However, educational levels were unevenly distributed across infection groups, χ² (1) = 5.13, *p* = .023, revealing a higher proportion of individuals with lower educational levels in the group previously infected (*n* = 71 with completed post-secondary education, *n* = 96 without completed post-secondary education) compared with the group previously not infected (*n* = 46 with completed post-secondary education, *n* = 32 without completed post-secondary education). For SSRT estimation, the integration method was employed, as it is more reliable and less biased than the mean method (see^ 143,156,160^). The integration method considers SSRT as the time point at which the integral of the response-time distribution equals the likelihood for incorrect responses in stop trials (see^143^). The time point at which the stopping process finishes thereby equals the *n*th response time, where *n* is the number of all go-trial responses (including choice errors and responses made before the stop signal occurred) multiplied by the likelihood of responding in a stop trial. To account for missing response latencies in omission-error go trials, maximum response latency was assigned to these trials (see^143^). SSRTs were log-transformed for regression analyses to meet the requirement of homoscedasticity.

## Electronic supplementary material

Below is the link to the electronic supplementary material.

Supplementary Material 1

## Data Availability

All datasets generated and/or analyzed within the current study are available from the corresponding author upon reasonable request.
