# Supplementary material for: Insights on the neurocognitive mechanisms underlying hippocampus-dependent memory impairment in COVID-19
Source: Sci Rep. 2025 Jun 20;15:20114. doi: 10.1038/s41598-025-04166-2 (PMC12181319; doi:10.1038/s41598-025-04166-2)
Supplement: Supplementary file 1 — Supplementary Material 1 [file 41598_2025_4166_MOESM1_ESM.docx]

**Supplementary information**

to the manuscript

“Insights on the Neurocognitive Mechanisms Underlying Hippocampus-Dependent Memory Impairment in COVID-19”

by Patric Meyer and Ann-Kathrin Zaiser

**Supplementary Table S1**

*Linear regression models to predict response latencies and accuracy in the Alertness task*

| Variable | *b* | SE | β | *t* | *p* |
| --- | --- | --- | --- | --- | --- |
| **Response Latencies (in s)** (*R*² = .08, adjusted *R*² = .03, *F*(7, 139) = 1.62, *p* = .134) | | | | | |
| Intercept | 0.36 | .01 |  | 41.58 | <.001*** |
| Infection status^a^ | 0.01 | .01 | .12 | 1.43 | .156 |
| Age | <.01 | <.01 | .15 | 1.73 | .086^†^ |
| Gender^b^ | -0.01 | .01 | -.16 | -1.85 | .066^†^ |
| Level of education^c^ | <.01 | .01 | .02 | 0.20 | .840 |
| Depressiveness | <.01 | <.01 | .11 | 0.80 | .425 |
| Anxiety | <.01 | <.01 | .15 | 1.02 | .311 |
| Stress | -<.01 | <.01 | -.24 | -1.63 | .104 |
| **Accuracy (***R*² = .05, adjusted *R*² = <.01, *F*(7, 141) = 1.03, *p* = .411) | | | | | |
| Intercept | 0.99 | <.01 |  | 335.81 | < .001*** |
| Infection status^a^ | <.01 | <.01 | .02 | 0.25 | .803 |
| Age | <.01 | <.01 | .02 | 0.28 | .778 |
| Gender^b^ | -<.01 | <.01 | -.17 | -2.06 | .041* |
| Level of education^c^ | <.01 | <.01 | .07 | 0.83 | .410 |
| Depressiveness | -<.01 | <.01 | -.21 | -1.55 | .123 |
| Anxiety | <.01 | <.01 | .14 | 0.95 | .343 |
| Stress | <.01 | <.01 | .02 | 0.16 | .877 |

*Note.* All *p* values and significance levels are reported two-tailed.

^a^ Previously uninfected = 0, previously infected = 1. ^b^ Female = 0, male = 1. ^c^ Without post-secondary education = 0, with post-secondary education = 1.

^†^*p* < .10. **p* < .05. ****p* < .001.

**Supplementary Table S2**

*Linear regression models to predict forward and backward block span in the Corsi block tapping task*

| Variable | *b* | SE | β | *t* | *p* |
| --- | --- | --- | --- | --- | --- |
| **Forward block span** (*R*² = .18, adjusted *R*² = .16, *F*(7, 241) = 7.70, *p* < .001) | | | | | |
| Intercept | 5.43 | .20 |  | 27.26 | <.001*** |
| Infection status^a^ | -0.31 | .15 | -.12 | -2.01 | .045* |
| Age | -0.03 | <.01 | -.33 | -5.55 | <.001*** |
| Gender^b^ | 0.53 | .14 | .23 | 3.80 | <.001*** |
| Level of education^c^ | 0.06 | .13 | .03 | 0.44 | .661 |
| Depressiveness | <.01 | .02 | .01 | 0.13 | .899 |
| Anxiety | -0.04 | .03 | -.14 | -1.32 | .189 |
| Stress | 0.01 | .01 | .11 | 0.98 | .330 |
| **Backward block span** (*R*² = .09, adjusted *R*² = .06, *F*(7, 215) = 3.06, *p* = .004) | | | | | |
| Intercept | 5.16 | .21 |  | 24.36 | <.001*** |
| Infection status^a^ | -0.24 | .17 | -.10 | -1.44 | .151 |
| Age | -0.01 | .01 | -.12 | -1.75 | .082^†^ |
| Gender^b^ | 0.14 | .15 | .06 | 0.92 | .357 |
| Level of education^c^ | 0.33 | .14 | .16 | 2.38 | .018* |
| Depressiveness | -0.04 | .02 | -.19 | -1.79 | .076^†^ |
| Anxiety | -0.02 | .03 | -.08 | -0.71 | .481 |
| Stress | <.01 | .01 | .09 | 0.74 | .463 |

*Note.* All *p* values and significance levels are reported two-tailed.

^a^ Previously uninfected = 0, previously infected = 1. ^b^ Female = 0, male = 1. ^c^ Without post-secondary education = 0, with post-secondary education = 1.

^†^*p* < .10. **p* < .05. ****p* < .001.

**Supplementary Table S3**

*Linear regression models to predict accuracy and hit response latencies in the n-back task*

| Variable | *b* | SE | β | *t* | *p* |
| --- | --- | --- | --- | --- | --- |
| **Accuracy (d’)** (*R*² = .14, adjusted *R*² = .12, *F*(7, 302) = 7.22, *p* < .001) | | | | | |
| Intercept | 2.26 | .12 |  | 18.72 | < .001*** |
| Infection status^a^ | -0.01 | .09 | -.01 | -0.12 | .908 |
| Age | -0.02 | <.01 | -.29 | -5.28 | <.001*** |
| Gender^b^ | 0.14 | .09 | .08 | 1.56 | .121 |
| Level of education^c^ | 0.24 | .08 | .16 | 2.93 | .004** |
| Depressiveness | -0.03 | .01 | -.16 | -1.89 | .060^†^ |
| Anxiety | -0.01 | .02 | -.07 | -0.81 | .421 |
| Stress | <.01 | <.01 | .02 | 0.19 | .847 |
| **Response Latencies** (**in s)** (*R*² = .12, adjusted *R*² = .10, *F*(7, 302) = 5.70, *p* < .001) | | | | | |
| Intercept | 0.66 | .02 |  | 41.30 | <.001*** |
| Infection status^a^ | -0.01 | .01 | -.06 | -1.11 | .270 |
| Age | <.01 | <.01 | .32 | 5.79 | <.001*** |
| Gender^b^ | -0.02 | .01 | -.07 | -1.28 | .201 |
| Level of education^c^ | 0.01 | .01 | .06 | 1.09 | .275 |
| Depressiveness | <.01 | <.01 | .01 | 0.12 | .904 |
| Anxiety | <.01 | <.01 | .13 | 1.40 | .162 |
| Stress | -<.01 | <.01 | -.14 | -1.44 | .150 |

*Note.* All *p* values and significance levels are reported two-tailed.

^a^ Previously uninfected = 0, previously infected = 1. ^b^ Female = 0, male = 1. ^c^ Without post-secondary education = 0, with post-secondary education = 1.

^†^*p* < .10. ***p* < .01. ****p* < .001.

**Supplementary Table S4**

*Linear regression models to predict specific switch costs and mixing costs in the task-switching task*, separately for response latencies and error rates

| Variable | *b* | SE | β | *t* | *p* |
| --- | --- | --- | --- | --- | --- |
| **Specific Switch Costs (log-transformed Response Latencies, in s)**  *R*² = .02, adjusted *R*² = -.01, *F*(7,299) = 0.69, *p* = .683 | | | | | |
| Intercept | 0.26 | .03 |  | 9.19 | <.001*** |
| Infection status^a^ | -<.01 | .02 | -.01 | -0.20 | .844 |
| Age | -<.01 | <.01 | -.12 | -1.97 | .049* |
| Gender^b^ | .01 | .02 | .03 | 0.46 | .645 |
| Level of education^c^ | <.01 | .02 | .01 | 0.11 | .913 |
| Depressiveness | -<.01 | <.01 | -.07 | -0.71 | .476 |
| Anxiety | -<.01 | <.01 | -.01 | -0.06 | .955 |
| Stress | <.01 | <.01 | .07 | 0.63 | .527 |
| **Mixing Costs (log-transformed Response Latencies, in s)**  *R*² = .05, adjusted *R*² = .03, *F*(7,300) = 2.45, *p* = .019 | | | | | |
| Intercept | .25 | .03 |  | 7.39 | <.001*** |
| Infection status^a^ | -.02 | .02 | -.04 | -0.75 | .456 |
| Age | <.01 | <.01 | .17 | 2.94 | .004** |
| Gender^b^ | -.04 | .02 | -.09 | -1.56 | .120 |
| Level of education^c^ | -.02 | .02 | -.06 | -1.00 | .318 |
| Depressiveness | <.01 | <.01 | .02 | 0.17 | .869 |
| Anxiety | <.01 | <.01 | .10 | 0.92 | .359 |
| Stress | <.01 | <.01 | .01 | 0.12 | .901 |
| **Specific Switch Costs (Error Rates)**  *R*² = .02, adjusted *R*² = -.0004, *F*(7,300) = 0.98, *p* = .444 | | | | | |
| Intercept | .01 | .01 |  | 1.71 | .088^†^ |
| Infection status^a^ | -<.01 | .01 | -.01 | -0.17 | .863 |
| Age | <.01 | <.01 | .04 | 0.64 | .522 |
| Gender^b^ | .01 | .01 | .08 | 1.38 | .168 |
| Level of education^c^ | <.01 | .01 | .01 | 0.19 | .849 |
| Depressiveness | <.01 | <.01 | .16 | 1.71 | .089^†^ |
| Anxiety | -<.01 | <.01 | -.11 | -1.06 | .288 |
| Stress | <.01 | <.01 | .06 | 0.52 | .601 |
| **Mixing Costs (Error Rates)**  *R*² = .04, adjusted *R*² = .02, *F*(7,297) = 1.95, *p* = .062 | | | | | |
| Intercept | .01 | <.01 |  | 1.88 | .062^†^ |
| Infection status^a^ | <.01 | .01 | .02 | 0.35 | .723 |
| Age | <.01 | <.01 | .13 | 2.22 | .027* |
| Gender^b^ | -.01 | .01 | -.05 | -0.92 | .361 |
| Level of education^c^ | -.01 | .01 | -.14 | -2.37 | .019* |
| Depressiveness | <.01 | <.01 | .03 | 0.35 | .723 |
| Anxiety | <.01 | <.01 | .01 | 0.11 | .912 |
| Stress | -<.01 | <.01 | -.06 | -0.53 | .595 |

*Note.* All *p* values and significance levels are reported two-tailed.

^a^ Previously uninfected = 0, previously infected = 1. ^b^ Female = 0, male = 1. ^c^ Without post-secondary education = 0, with post-secondary education = 1.

^†^*p* < .10. **p* < .05. ***p* < .01. ****p* < .001.

**Supplementary Table S5**

*Linear regression models to predict accuracy and response latencies in the go/no-go task*

| Variable | *b* | SE | β | *t* | *p* |
| --- | --- | --- | --- | --- | --- |
| **Accuracy (d’)** (*Conditional R*² = .61, *marginal* *R*² = .13) | | | | | |
| Intercept | 3.63 | .13 |  | 28.09 | <.001*** |
| Infection status^a^ | .01 | .08 | .01 | 0.13 | .898 |
| Task phase^b^ | -.47 | .04 | -.29 | -11.21 | <.001*** |
| Age | .01 | <.01 | .13 | 2.60 | .010** |
| Gender^d^ | -.21 | .09 | -.11 | -2.33 | .020* |
| Level of education^e^ | .17 | .08 | .10 | 2.12 | .035* |
| Depressiveness | -.03 | .01 | -.18 | -2.24 | .026* |
| Anxiety | .02 | .02 | .08 | 0.97 | .333 |
| Stress | <.01 | <.01 | .01 | 0.07 | .948 |
| **Accuracy (Commission Errors)** (*Conditional R*² = .60, *marginal* *R*² = .21) | | | | | |
| Intercept | 0.07 | .03 |  | 2.61 | .009** |
| Infection status^a^ | -.01 | .02 | -.03 | -0.67 | .504 |
| Task phase^b^ | .13 | .01 | .36 | 13.71 | <.001*** |
| Age | -<.01 | <.01 | -.24 | -5.24 | <.001*** |
| Gender^d^ | .06 | .02 | .15 | 3.39 | <.001*** |
| Level of education^e^ | -.03 | .02 | -.07 | -1.62 | .107 |
| Depressiveness | <.01 | <.01 | .10 | 1.35 | .177 |
| Anxiety | -<.01 | <.01 | -.04 | -0.49 | .622 |
| Stress | <.01 | <.01 | .01 | 0.19 | .853 |
| **Response latencies (in s)** (*Conditional R*² = .65, *marginal R*² = .30) | | | | | |
| Intercept | .38 | .01 |  | 45.30 | <.001*** |
| Infection status^a^ | -<.01 | .01 | -.03 | -0.66 | .511 |
| Task phase^b^ | .02 | <.01 | .11 | 6.56 | <.001*** |
| Trial type^c^ | -.06 | <.01 | -.44 | -25.46 | <.001*** |
| Age | <.01 | <.01 | .30 | 7.77 | <.001*** |
| Gender^d^ | -<.01 | .01 | -.02 | -0.42 | .673 |
| Level of education^e^ | <.01 | .01 | <.01 | 0.06 | .954 |
| Depressiveness | <.01 | <.01 | .09 | 1.40 | .162 |
| Anxiety | <.01 | <.01 | .06 | 0.86 | .392 |
| Stress | -<.01 | <.01 | -.13 | -1.80 | .073^†^ |

*Note.* All *p* values and significance levels are reported two-tailed.

^a^ Previously uninfected = 0, previously infected = 1. ^b^ Phase 1 = 1, Phase 2 = 2. ^c^ Go trials = 0, no-go trials = 1. ^d^ Female = 0, male = 1. ^e^ Without post-secondary education = 0, with post-secondary education = 1.

^†^*p* < .10. **p* < .05. ***p* < .01. ****p* < .001.

**Supplementary Table S6**

*Linear regression model to predict stop-signal reaction time (SSRT) in the stop-signal task*

| Variable | *b* | SE | β | *t* | *p* |
| --- | --- | --- | --- | --- | --- |
| **Log-transformed Stop-Signal Reaction Time (SSRT; in s)**  *R*² = .10, adjusted *R*² = .07, *F*(7, 237) = 3.81, *p* < .001 | | | | | |
| Intercept | -0.53 | .04 |  | -14.82 | <.001*** |
| Infection status^a^ | -<.01 | .03 | -.01 | -0.15 | .880 |
| Age | <.01 | <.01 | .29 | 4.55 | <.001*** |
| Gender^b^ | 0.01 | .03 | .02 | 0.27 | .791 |
| Level of education^c^ | 0.02 | .02 | .04 | 0.70 | .487 |
| Depressiveness | -<.01 | <.01 | -.11 | -1.12 | .263 |
| Anxiety | .01 | .01 | .16 | 1.42 | .156 |
| Stress | -<.01 | <.01 | -.08 | -0.78 | .438 |

*Note.* All *p* values and significance levels are reported two-tailed.

^a^ Previously uninfected = 0, previously infected = 1. ^b^ Female = 0, male = 1. ^c^ Without post-secondary education = 0, with post-secondary education = 1.

****p* < .001.
